# The duration of chemoprophylaxis against malaria after treatment with artesunate-amodiaquine and artemether-lumefantrine and the effects of *pfmdr1* 86Y and *pfcrt* 76T: a meta-analysis of individual patient data

**DOI:** 10.1186/s12916-020-1494-3

**Published:** 2020-02-25

**Authors:** Michael T. Bretscher, Prabin Dahal, Jamie Griffin, Kasia Stepniewska, Quique Bassat, Elisabeth Baudin, Umberto D’Alessandro, Abdoulaye A. Djimde, Grant Dorsey, Emmanuelle Espié, Bakary Fofana, Raquel González, Elizabeth Juma, Corine Karema, Estrella Lasry, Bertrand Lell, Nines Lima, Clara Menéndez, Ghyslain Mombo-Ngoma, Clarissa Moreira, Frederic Nikiema, Jean B. Ouédraogo, Sarah G. Staedke, Halidou Tinto, Innocent Valea, Adoke Yeka, Azra C. Ghani, Philippe J. Guerin, Lucy C. Okell

**Affiliations:** 1 MRC Centre for Global Infectious Disease Analysis, Department of Infectious Disease Epidemiology, Imperial College London, London, UK; 2 WorldWide Antimalarial Resistance Network (WWARN), Oxford, UK; 3 Centre for Tropical Medicine & Global Health, Nuffield Department of Medicine, University of Oxford, Oxford, UK; 4 School of Mathematical Sciences, Queen Mary University of London, London, UK; 5 Centro de Investigação em Saúde de Manhiça (CISM), Maputo, Mozambique; 6 ISGlobal, Hospital Clínic - Universitat de Barcelona, Barcelona, Spain; 7 ICREA, Pg. Lluís Companys 23, 08010 Barcelona, Spain; 8 Pediatric Infectious Diseases Unit, Pediatrics Department, Hospital Sant Joan de Déu (University of Barcelona), Barcelona, Spain; 9 Consorcio de Investigación Biomédica en Red de Epidemiología y Salud Pública (CIBERESP), Madrid, Spain; 10 Epicentre, Paris, France; 11 MRC Unit The Gambia at the London School of Hygiene and Tropical Medicine, Fajara, The Gambia; 12 Malaria Research and Training Center, University of Science, Techniques and Technologies of Bamako, Bamako, Mali; 13 Department of Medicine, University of California San Francisco, San Francisco, USA; 14 Clinical and Epidemiology Department, GSK Vaccines, R&D Center, Wavre, Belgium; 15 Centre for Global Health Research, Kenya Medical Research Institute, Kisumu, Kenya; 16 Swiss Tropical and Public Health Institute, Basel, Switzerland; 17 University of Basel, Basel, Switzerland; 18 Medecins Sans Frontieres-OCBA, Barcelona, Spain; 19 Department of Medicine I, Division of Infectious Diseases and Tropical Medicine, Medical University of Vienna, Vienna, Austria; 20 Centre de Recherches Medicales de Lambarene, Lambarene, Gabon; 21 Department of Paediatrics, University of Calabar, Calabar, Nigeria; 22 Institute for Tropical Medicine, University of Tubingen, Tubingen, Germany; 23 Department of Tropical Medicine, Bernhard Nocht Institute for Tropical Medicine and I. Department of Medicine, University Medical Center Hamburg-Eppendorf, Hamburg, Germany; 24 Institut de Recherche en Science de la Sante, Bobo-Dioulasso, Burkina Faso; 25 Department of Clinical Research, Faculty of Infectious & Tropical Diseases, London School of Hygiene & Tropical Medicine, London, UK; 26 Institut de Recherche en Science de la Sante, Nanoro, Burkina Faso; 27 Uganda Malaria Surveillance Project, Kampala, Uganda

**Keywords:** Malaria, Artemisinin, Drug, Lumefantrine, Amodiaquine, Trial, Mathematical model, *mdr1*, *Crt*

## Abstract

**Background:**

The majority of *Plasmodium falciparum* malaria cases in Africa are treated with the artemisinin combination therapies artemether-lumefantrine (AL) and artesunate-amodiaquine (AS-AQ), with amodiaquine being also widely used as part of seasonal malaria chemoprevention programs combined with sulfadoxine-pyrimethamine. While artemisinin derivatives have a short half-life, lumefantrine and amodiaquine may give rise to differing durations of post-treatment prophylaxis, an important additional benefit to patients in higher transmission areas.

**Methods:**

We analyzed individual patient data from 8 clinical trials of AL versus AS-AQ in 12 sites in Africa (*n* = 4214 individuals). The time to PCR-confirmed reinfection after treatment was used to estimate the duration of post-treatment protection, accounting for variation in transmission intensity between settings using hidden semi-Markov models. Accelerated failure-time models were used to identify potential effects of covariates on the time to reinfection. The estimated duration of chemoprophylaxis was then used in a mathematical model of malaria transmission to determine the potential public health impact of each drug when used for first-line treatment.

**Results:**

We estimated a mean duration of post-treatment protection of 13.0 days (95% CI 10.7–15.7) for AL and 15.2 days (95% CI 12.8–18.4) for AS-AQ overall. However, the duration varied significantly between trial sites, from 8.7–18.6 days for AL and 10.2–18.7 days for AS-AQ. Significant predictors of time to reinfection in multivariable models were transmission intensity, age, drug, and parasite genotype. Where wild type *pfmdr1* and *pfcrt* parasite genotypes predominated (<=20% 86Y and 76T mutants, respectively), AS-AQ provided ~ 2-fold longer protection than AL. Conversely, at a higher prevalence of 86Y and 76T mutant parasites (> 80%), AL provided up to 1.5-fold longer protection than AS-AQ. Our simulations found that these differences in the duration of protection could alter population-level clinical incidence of malaria by up to 14% in under-5-year-old children when the drugs were used as first-line treatments in areas with high, seasonal transmission.

**Conclusion:**

Choosing a first-line treatment which provides optimal post-treatment prophylaxis given the local prevalence of resistance-associated markers could make a significant contribution to reducing malaria morbidity.

## Background

Nearly all malaria-endemic countries use artemisinin-based combination therapies (ACTs) as first-line treatment for uncomplicated *Plasmodium falciparum* malaria. In each ACT, the artemisinin derivative is combined with a different antimalarial partner drug. There are currently five ACTs recommended by the World Health Organization (WHO): artemether-lumefantrine (AL), artesunate-amodiaquine (AS-AQ), dihydroartemisinin (DHA)-piperaquine, artesunate-mefloquine, and artesunate-sulfadoxine-pyrimethamine (AS-SP) [1]. In areas where other ACTs are failing, WHO also suggest considering a sixth ACT: artesunate-pyronaridine, now prequalified by WHO [2].

Each of the six drug regimens has different pharmacokinetic and pharmacodynamic properties, and these have implications for the public health benefit of the drugs in terms of their ability to reduce overall malaria transmission in the community, as well as cure disease [3]. The artemisinin derivatives are highly potent antimalarials that rapidly reduce the parasite biomass; however, they have a very short half-life. The partner drugs remain in the blood for longer, clearing remaining parasites and incidentally providing chemoprophylaxis against reinfection which may have an important impact in moderate-to-high transmission areas [4–6]. Some antimalarials have additional activity against gametocytes, the transmissible form of the parasite, and these are better at preventing onward transmission from the patient after treatment. Gametocyte killing may therefore benefit the community through reduction of the overall transmission level [5].

Artemether-lumefantrine (AL) is globally the most widely used ACT, followed by artesunate-amodiaquine (AS-AQ) [7]. While resistance to artemisinin has emerged in South-East Asia [8] and a degree of resistance to the partner drugs exists in some parts of the world, both treatments remain highly effective in most African malaria-endemic areas [9–12]. The pharmacokinetic properties of each drug are relatively well characterized: lumefantrine and its metabolite desbutyl-lumefantrine have terminal elimination half-lives of 1–10 days [1, 13–16], while desethylamodiaquine, the active metabolite of amodiaquine, has a half-life of 4–10 days [1, 17–22]. However, these estimates do not provide information on the duration of post-treatment prophylaxis which also depends on the pharmacodynamics of the drug.

There is evidence that the duration of protection after AS-AQ and AL treatment is affected by parasite mutations associated with reduced drug sensitivity [9, 11]. These two drugs show collateral sensitivity, such that the mutations 86Y and 1246Y in the *P. falciparum multidrug resistance transporter 1* (*pfmdr1)* gene and 76T in the *P. falciparum chloroquine resistance transporter (pfcrt)* gene are linked to reduced sensitivity to AS-AQ but increased sensitivity to AL, which is thought to be due to differential sensitivity to the amodiaquine and lumefantrine partner drugs rather than the artemisinin. Although the overall efficacy of each drug remains high in Africa, a meta-analysis found that the N86 wild type parasite was associated with a fourfold increased risk of recrudescence after AL treatment [9, 11]. All these mutations were also associated with a reduced time to reinfection after AS-AQ treatment, and an increased time to reinfection after AL treatment, although the exact duration of protection was not estimated since this also depends on the local rate of transmission and thus reinfection.

The duration of protection can be estimated from clinical trials where reinfection rates are monitored. We previously estimated the mean protection provided by AL at 13.8 days, and DHA-piperaquine at 29.4 days [4]. The duration of protection provided by amodiaquine is not well known, although there are indications that it might confer longer protection than lumefantrine [23, 24]. Here, we use a statistical analysis of pooled clinical trial data from multiple sites in Africa, explicitly incorporating local transmission intensity as well as drug effects into analyzing the time to reinfection, to estimate the duration of post-treatment prophylaxis after AS-AQ and AL. We use these results in an epidemiological transmission model to establish the differences in public health impact when AS-AQ versus AL is used as first-line drug for *P. falciparum* case management.

## Methods

### Overview

To assess the duration of post-treatment prophylaxis provided by AL and AS-AQ, we analyzed clinical trial data obtained from the WorldWide Antimalarial Resistance Network (WWARN) data sharing platform [25] with the consent of study authors. Two statistical approaches were employed: a hidden semi-Markov model allowed for estimation of the actual duration of chemoprophylaxis (which is shorter than the time to reinfection), and accelerated failure-time models provided a better understanding of the factors that modify it. Finally, we used a mathematical model to simulate the epidemiological consequences of using AS-AQ or AL as first-line antimalarial drugs.

### Data

WWARN invited investigators to contribute individual-level patient data for this meta-analysis [26] if their studies fulfilled the following criteria: randomized controlled trials of uncomplicated *P. falciparum* malaria; AS-AQ and AL being compared; follow-up to at least day 28, with at least one follow-up visit at day 14 and another before day 28; 100 or more participants per study site or more than 28 days follow-up; polymerase chain reaction (PCR)-adjusted efficacy available; at least 95% PCR-adjusted treatment efficacy in both study arms; PCR-unadjusted cure rates of < 95% in at least one trial arm by day 28 (to indicate sufficient number of reinfections to inform analysis on post-treatment prophylaxis); standard dose regimens of AL and AS-AQ (we included studies regardless whether AS-AQ was given as a fixed-dose combination or not); and known dosage taken for each patient. Individual patient data from eligible studies were shared, collated, and standardized using previously described methodology [27].

For the present analyses, we used data on PCR-confirmed reinfections as well as the proportion of patients who were not reinfected during follow-up, to estimate the duration of chemoprophylaxis. Time of reinfection is included in the analysis so that different follow-up times between studies are accounted for (see also below). Patients who experienced PCR-confirmed recrudescence were excluded. The majority of included trials did PCR correction using three molecular markers: *glurp*, *msp1*, and *msp2* (Table 1). We also did a sensitivity analysis to explore the possibility that some of the recrudescences identified by this PCR correction method could have been misclassified as reinfections. Recent work suggests that the percentage of patients experiencing recrudescence may be around 1–3% higher than estimated by standard PCR correction [64–66], with this error being relatively constant across transmission settings. We therefore also repeated our analysis after reclassifying some reinfections in each trial as recrudescences, sampling a number that would achieve a 3% higher recrudescence rate overall. We weighted the sampling by timing of recurrent parasitemia in each patient as in Fig. 5 of [66], i.e., to allow for the fact that recrudescences are more likely to occur early during follow-up (see also Additional file 4: Figure S3 legend).

**Table 1:** Clinical trials included in the analysis and fitted parameters for each trial. The study sites are shown in order of increasing transmission intensity, as estimated by the hidden semi-Markov model analysis. Prior EIRs are estimated from the Malaria Atlas Project slide prevalence for each location in the year of the trial [28, 29]

Site and reference	Country, year	PCR correction: molecular markers	*N* (AL/AS-AQ)	AS-AQ manufacturer (formulation), target AQ dose*	Days of prophylaxis: posterior median (95% CI)		EIR		FOI^†^	Prevalence of *pfmdr1* 86Y, % (references)	Prevalence of *pfcrt* 76T, % (references)
					AL	AS-AQ	Prior mean	Posterior median (95% CI)			
Fougamou [23]	Gabon, 2007–2008	*msp1*,*msp2*,*glurp*	68/68	Sanofi-Aventis (FDC Coarsucam) 30 mg/kg	11.6 (6.0–16.8)	13.1 (7.6–18.6)	0.6	2.3 (1.1–4.2)	0.5	79.5 [30]	97.9 [31]
Ndola [23]	Zambia, 2007–2009	*msp1*,*msp2*,*glurp*	69/64	Sanofi-Aventis (FDC Coarsucam) 30 mg/kg	10.8 (6.0–14.8)	16.1 (9.7–25.0)	1.2	4.8 (2.4–8.4)	1.0	No matching survey	20.8 [32]
Pweto [33]	Democratic Republic of Congo 2008-2009	*msp1*,*msp2*,*glurp*	126/129	Sanofi-Aventis (AS-AQ Winthrop FDC) 30 mg/kg	11.3 (7.8–14.4)	17.9 (12.1–25.6)	50.0	9.3 (6.2–13.7)	2.0	No matching survey	No matching survey
Pamol [23]	Nigeria, 2007–2008	*msp1*,*msp2*,*glurp*	164/159	Sanofi-Aventis (FDC Coarsucam) 30 mg/kg	17.9 (12.3–22.5)	15.4 (10.3–21.7)	22.6	9.5 (4.7–21.6)	2.2	61.8 [34–36]	90.1 [37]
Bobo Dioulasso (unpublished‡)	Burkina Faso, 2010–2012	*msp1*,*msp2*	373/372	Sanofi-Aventis (FDC Coarsucam) 30 mg/kg	12.5 (10.6–14.4)	16.9 (14.0–19.9)	21.5	17.4 (13.3–23.1)	5.9	18.0 [38, 39]	28.5 [38–42]
Gourcy (unpublished‡)	Burkina Faso, 2010–2012	*msp1*,*msp2*	112/129	Sanofi-Aventis (FDC Coarsucam) 30 mg/kg	8.7 (6.2–10.9)	17.8 (13.7–22.1)	22.9	23.3 (15.9–33.7)	6.5	18.0 [38, 39]	24.8 [39, 41]
Kisumu (unpublished‡)	Kenya, 2005	*msp1*,*msp2*	179/178	Sanofi and Hoechst Marion Roussel (Loose NFDC) 30 mg/kg	18.6 (15.8–21.2)	14.2 (10.9–17.6)	6.9	26.5 (18.1–39.6)	5.7	66.6 [43–46]	90.3 [44, 46, 47]
Nimba [48]	Liberia, 2008–2009	*msp1*,*msp2*,*glurp*	127/141	Sanofi-Aventis (AS-AQ Winthrop FDC) 30 mg/kg	17.9 (15.1–20.6)	11.6 (8.8–14.3)	18.3	32.4 (26.1–40.0)	8.0	69.4 [49]	93.5 [49]
Sikasso [50]	Mali 2005-2007	*msp1*,*msp2*,*CA1*	236/233	Sanofi-Aventis (Coblistered NFDC. Arsucam) 30 mg/kg	10.2 (9.0–11.6)	18.7 (16.1–21.5)	25.2	37.2 (29.5–46.9)	11.3	35.5 [51]	70.2 [32, 51–54]
Tororo [55]	Uganda, 2009–2010	*msp1*,*msp2*,*glurp*	190/190	Sanofi (AS-AQ Winthrop FDC) 30 mg/kg	13.3 (11.8–14.6)	13.4 (11.7–15.1)	23.3	84.2 (72.9–96.9)	16.9	63.9 [56–58]	99.6 [56, 58]
Nanoro [23]	Burkina Faso, 2007–2008	*msp1*,*msp2*,*glurp*	257/273	Sanofi-Aventis (FDC Coarsucam) 30 mg/kg	10.1 (9.2–11.1)	17.0 (15.0–19.2)	52.2	91.9 (76.2–111.1)	18.9	31.6 [38, 59]	67.0 [32, 38, 42, 59]
Tororo [60]	Uganda, 2005	*msp1*,*msp2*	189/195	AQ: Parke-David, Pfizer, AS: Sanofi-Aventis (Loose NFDC) 25 mg/kg	12.4 (11.1–13.8)	10.2 (8.9–11.6)	64.6	117.1 (98.4–139.8)	23.3	79.4 [56, 61]	96.2 [62, 63]

*FDC fixed-dose combination, *NFDC* non-fixed-dose combination. AS-AQ FDC was from Sanofi. For AL, all trials used the Novartis fixed-dose combination and the same dose regimen

^†^*FOI* force of infection, estimated mean incidence of patent blood-stage infection in this trial population, given the age distribution and fitted EIR

‡ Unpublished study references: Bobo Dioulasso, Gourcy: Nikiema F, Zongo I, Some F, Ouedraogo J. Evolution of therapeutic efficacies of artemisinin-based combination therapies (ASAQ and AL) for treatment of uncomplicated falciparum malaria in Burkina Faso during five years of adoption as first-line treatments, unpublished. and Kisumu: Juma EA. Efficacy of co-administered amodiaquine plus artesunate and artemether/lumefantrine for the treatment of uncomplicated falciparum malaria in children less than five years in different epidemiological settings in Kenya, unpublished.

In two studies (in Tororo, Uganda and Sikasso, Mali, see Table 1), the patients were followed up longitudinally across several episodes and consequently treated multiple times within short intervals. We only used the first treatment episode and follow-up data collected before the next episode from these studies in order to avoid confounding of our results by residual drug levels from a previous treatment. One included study did not have available data on the individual ages of participants, but provided body weight [55], and another study recorded age but not body weight [50]. We imputed the missing values in order to be able to include these studies. To impute missing age, we randomly sampled ages of participants of the same gender from all other studies who had body weights within 0.5 kg of the observed participants’ weights; to impute missing body weight, we sampled weights of individuals of the same gender within 0.5 years of age for those under 25, and within 5 years for those over 25 years of age.

Molecular markers associated with susceptibility to AL and AS-AQ were not directly measured during these trials. Instead, for each trial, we sought other studies close in space and time which measured the prevalence of *pfcrt* 76T, *pfmdr1* 86Y, and *pfmdr1* 1246Y mutations among infected individuals, using recently completed systematic reviews [67, 68]. We included matches when the study was conducted in the same country, within 300 km of the trial site and within 1 year of the trial start or end year. When more than one matching survey was found, we took a weighted average of the mutant prevalence. For sites with many matching molecular marker surveys, we applied a stricter distance criterion of 100 km of the trial site. We did not include molecular marker studies on post-treatment samples.

### Prior information on the entomological inoculation rate (EIR)

The time to reinfection in these trials is only in part determined by the duration of protection conferred by the drug. This is because individuals do not immediately become reinfected after the protection ends, but rather enter an “at-risk” state. Thereafter, they are reinfected at a rate dependent on the incidence of patent blood-stage infections in the population (the force of infection (FOI) which in turn depends on the entomological inoculation rate (EIR), the number of infectious bites per person per year). More specifically, the time span between the end of the protected period and reinfection follows an exponential distribution with mean 1/*φ*, assuming a time-constant FOI *φ*. We used predictions of the EIR as prior values in our model, based on prevalence of infection in 2–10-year-olds estimated by the Malaria Atlas Project at the location and year in which each trial was carried out [28, 29]. When the trial took place over several years, we averaged slide prevalence over this time. These prevalence values were transformed into predictions of the EIR and FOI using the relationships obtained from our existing mathematical model of malaria transmission [69], which has a fixed relationship between EIR and FOI for a given age and history of exposure, allowing calculation of location-specific prior values for φ as explained below.

### Hidden semi-Markov models

The transition of an individual from a drug-protected state to a non-protected state, where they are at risk of reinfection after chemoprophylaxis, is not observed. We observe only whether the patient has become reinfected, after a certain time has passed since treatment. This sequence of events can be interpreted as realization of a stochastic process belonging to the class of hidden semi-Markov models, which we used to estimate the duration of protection provided by treatment. More specifically, we modeled the time to reinfection *R*_*i*_ in host *i* as

$$ {R}_i={P}_{di}+{I}_i+\delta $$

where *P*_*di*_ is the duration of chemoprophylaxis of drug *d* in host *i*, *I*_*i*_ is the time until reinfection occurs in host *i* once at risk, and *δ* represents the time required for a blood-stage infection to become patent after hepatocyte rupture (assumed 3.5 days [71]). *P* and *I* were parameterized as random variables as follows:

$$ {P}_{di}\sim \mathrm{gamma}\left({\lambda}_{di},{r}_d\right) $$

where the drug-specific scale parameter *λ* and shape parameter *r* are to be estimated, and

$$ {I}_i\sim \exp \left(1/{\varphi}_i\right) $$

with φ_*i*_ being the force of infection to which individual *i* was exposed during the trial follow-up. We assume that protection by the drug is all-or-nothing and that protection times in the population follow a gamma distribution, with a median for each drug that is constant in each trial site. The variance of this gamma distribution incorporates the effect of factors that are not specifically modeled, such as variation in pharmacokinetics, and potentially variation in sensitivity of different parasite clones to the drugs within each site. Individual-specific EIR values *ε*_*i*_ were determined, taking into account that young children are bitten less often due to their smaller body size, according to the formula

$$ {\varepsilon}_i={\varepsilon}_{\mathrm{adult}}\ \left(1-\rho\ \exp \left(-{a}_i/{a}_0\right)\right) $$

where *ε*_adult_ is the estimated site-specific EIR experienced by fully grown individuals, *a* is age and parameters *a*_0_ = 2920 days and *ρ* = 0.85 control the shape of the relationship [71]. Pre-erythrocytic immunity, i.e., an immune response that reduces the proportion of infectious bites resulting in successful blood-stage infections, was computed for each individual according to their age, prior exposure and local EIR, using the same mathematical model referenced above [69]. Both age-related biting and pre-erythrocytic immunity were therefore fixed for each individual based on their age and the local EIR based on this previous work. For sensitivity analysis, we also tried assuming additional age-independent variation in exposure to mosquito bites, with the distribution of relative biting rates across people following a lognormal distribution. We used informative priors on the lognormal distribution of bites of mean = 1 and variance = 1.76 because these have been previously estimated [69].

A number of hidden semi-Markov model variants were fitted via MCMC (Markov-Chain Monte Carlo), using the JAGS (“Just Another Gibbs Sampler”) software for Bayesian inference in conjunction with the “rjags” package using R statistical software [72]. The likelihood calculation took into account the interval- and right-censoring of observations in the data. EIR values *ε*_adult_ for each site were estimated simultaneously with the other parameters, with moderately informative gamma priors with median as predicted by the Malaria Atlas Project [29] (Table 1) and a shape parameter of 1.56. Using this prior information on EIR was essential; otherwise, a slow reinfection rate could be explained equally well by either a low EIR or a long drug prophylactic time. The shape parameter of the EIR priors was chosen to achieve a compromise between being giving a flexible enough prior that the model could fit the data in each site, to allow for seasonal variations and uncertainties in Malaria Atlas Project EIR estimates, while not allowing a systematic drift of posterior EIR estimates to be all lower or higher than the priors. A prior shape parameter much lower than 1.5, giving a less informative prior, produced poor convergence of EIR MCMC chains, and at any one point in the chain, the EIR estimates could drift to either be all lower or all higher than the prior medians with a corresponding decrease or increase in the estimated prophylactic times. We considered that such a systematic error in the estimates based on the Malaria Atlas Project would be unlikely. After examining the posterior distributions of several candidate models, we included heterogeneity among trial sites in the mean duration of chemoprophylaxis, which was modeled as a gamma-distributed random effect. A weakly informative, empirical-Bayes gamma prior was used for the shape parameter *r*, with hyperparameters (parameters of the prior distribution) determined using a fit of the hidden semi-Markov model with non-informative priors. This improved MCMC convergence. Non-informative gamma priors were chosen for all remaining estimated parameters. We ran the MCMC procedure for 1.25 million iterations, retaining 100,000 samples of the posterior after discarding 4000 adaptation steps, 4000 burn-in steps, and thinning.

### Accelerated failure-time models

In order to identify which factors influence the time until a reinfection is detected, we used accelerated failure-time models, as implemented in the “survival” package in R [73]. We explored lognormal and log-logistic distributions of time to reinfection, which allow the hazard of reinfection to vary over time, and selected lognormal which produced lowest Akaike Information Criterion (AIC). Several covariates were compared with respect to their ability to predict time to reinfection. Since EIR is such a critical predictor of the time to reinfection, we adjusted for this variable in all models, initially in bivariate models with each other covariate, using the log posterior mean EIR estimates from the hidden semi-Markov model analysis for each site. When analyzing age as a covariate, we explored polynomial relationships with reinfection time. The small proportion of individuals in the analysis over 20 years of age (294/3840 with available age data) were grouped together, since model convergence problems were created by lack of data at older ages and because age-dependent exposure to mosquito bites (related to body surface area) [74], as well as development of immunity [69], tends to plateau by 20 years of age. Otherwise, linear relationships were assumed for continuous variables. We tested for interactions between AL and AS-AQ treatment, prevalence of the *pfmdr1* 86Y mutant versus N86 wild type parasites, and *pfcrt* 76T mutant versus K76 wild type parasites, since there is evidence of differential effects of each drug on these parasite genotypes [9, 11]. We tested for an effect of different formulations of AS-AQ, i.e., fixed-dose combination (from Sanofi), blister pack, or loose dose (see also Table 1 for dose information). For AL, all included studies used the same fixed-dose combination from Novartis. We calculated weight-for-age *Z* scores for patients under 5 years old according to the WHO age- and gender-specific reference values, using the WHO Anthro software in R [75]. Individuals were classified as underweight if they had a *Z* score of less than − 2. We investigated being underweight in the children under 5 years because this was a factor associated with recrudescence after AL in a previous analysis [10]. We calculated milligram per kilogram dose of lumefantrine or amodiaquine for each patient according to their dose and weight. Goodness of fit of the models was assessed by AIC. We used stepwise regression, with both forward selection and backward elimination to ensure all covariates of interest were identified. The best-fitting model was identified using AIC, and covariates significantly improving the prediction (likelihood ratio test) were kept.

### Epidemiological simulations

An existing mathematical model of *Plasmodium falciparum* epidemiology [69] was used to assess the impact of first-line antimalarial treatment on malaria transmission outcomes. The model incorporates clinical episodes by age and exposure and has been fitted to data in a wide variety of settings [69].We included the results of the hidden semi-Markov model analysis on the distribution of protection times of AL and AS-AQ in the model. The model was first run to equilibrium in the absence of interventions, then we simulated first-line treatment with AS-AQ or AL, assuming that 80% of clinical episodes are treated with an antimalarial, that both drugs are 95% efficacious at clearing parasites, and that the switch is instantaneous and complete. Prior to introducing ACT, we assume SP was in use, also at 80% coverage but only 60% efficacy. We simulated a population of 600,000 individuals to smooth stochastic variation. We adjusted mosquito densities to represent low, medium, and high transmission areas with and without seasonal variation (pre-intervention slide prevalence in 2–10-year-olds = 5%, 15%, and 50%, respectively in the non-seasonal settings). In seasonally varying settings (Additional file 2: Figure S1), we set the annual EIR to be the same as in each respective low, medium, or high transmission non-seasonal setting. The probability of a mosquito becoming infected when feeding on individuals treated with AL relative to untreated individuals was assumed to be 0.051 [69]. It is uncertain whether there is any difference in human infectiousness after treatment with AL versus AS-AQ. We therefore ran the simulations twice, assuming firstly that patients are equally infectious after treatment with either ACT, and assuming secondly that patients treated with AS-AQ are twice as infectious, in approximate accordance with the ratio of areas under curves of post-treatment gametocyte prevalence in Schramm et al. [48] which is consistent with a meta-analysis showing reduced gametocytemia after treatment with AL compared with AS-AQ [76].

## Results

### Duration of protection after AL and AS-AQ treatment in different trial sites

**Fig. 1:**
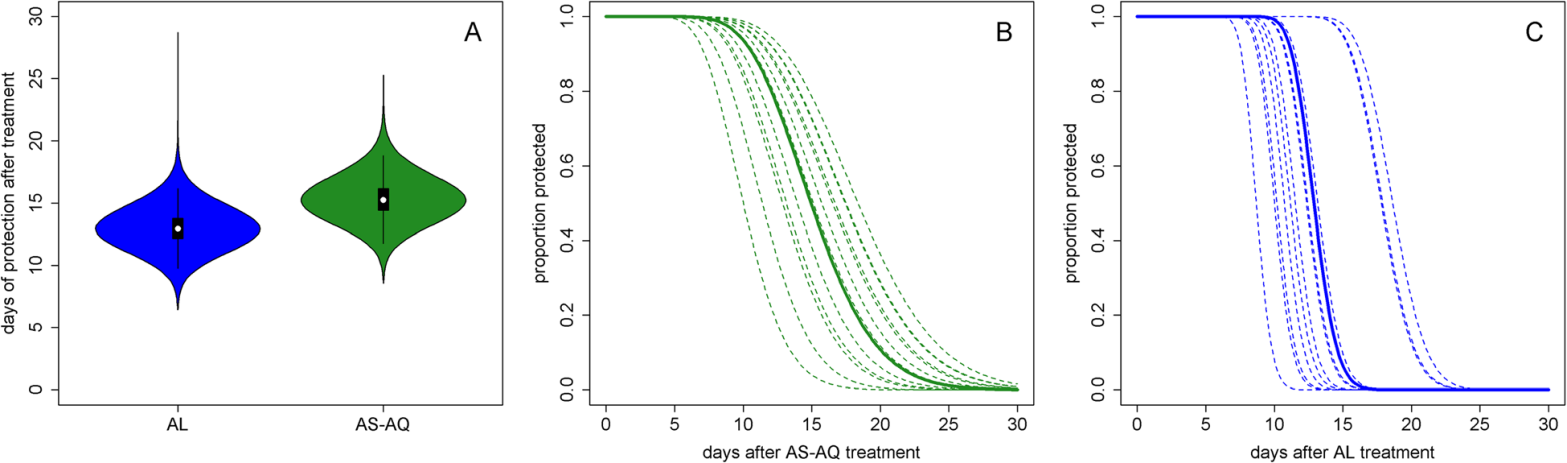
Duration of post-treatment prophylaxis. Posterior estimates of the median duration of protection (**a**) and the proportion of the population still having drug levels which would protect them from reinfection, over time since first dose with either AS-AQ (**b**) or AL (**c**). In **b** and **c**, the solid lines show the median estimate across trial sites, while the dotted lines show the different estimates for each of the 12 trial sites. The equations of the lines in **b** and **c** are reverse cumulative gamma distributions and can be implemented for example in R as 1-pgamma(*t*, shape = *r*, scale = *λ*), where *t* is time in days, and *r* and *λ* are the shape and scale parameters of the gamma distribution, respectively. For AL, *r* = 93.5 and mean *λ* = 0.139. For AS-AQ, *r* = 16.8 and mean *λ* = 0.906. The mean of each gamma distribution *rλ* gives the duration of protection from each drug. The site-specific lines can be calculated using the median durations of prophylaxis in Table 1, and the same shape parameter (assumed not to vary between sites for each drug)

**Fig. 2:**
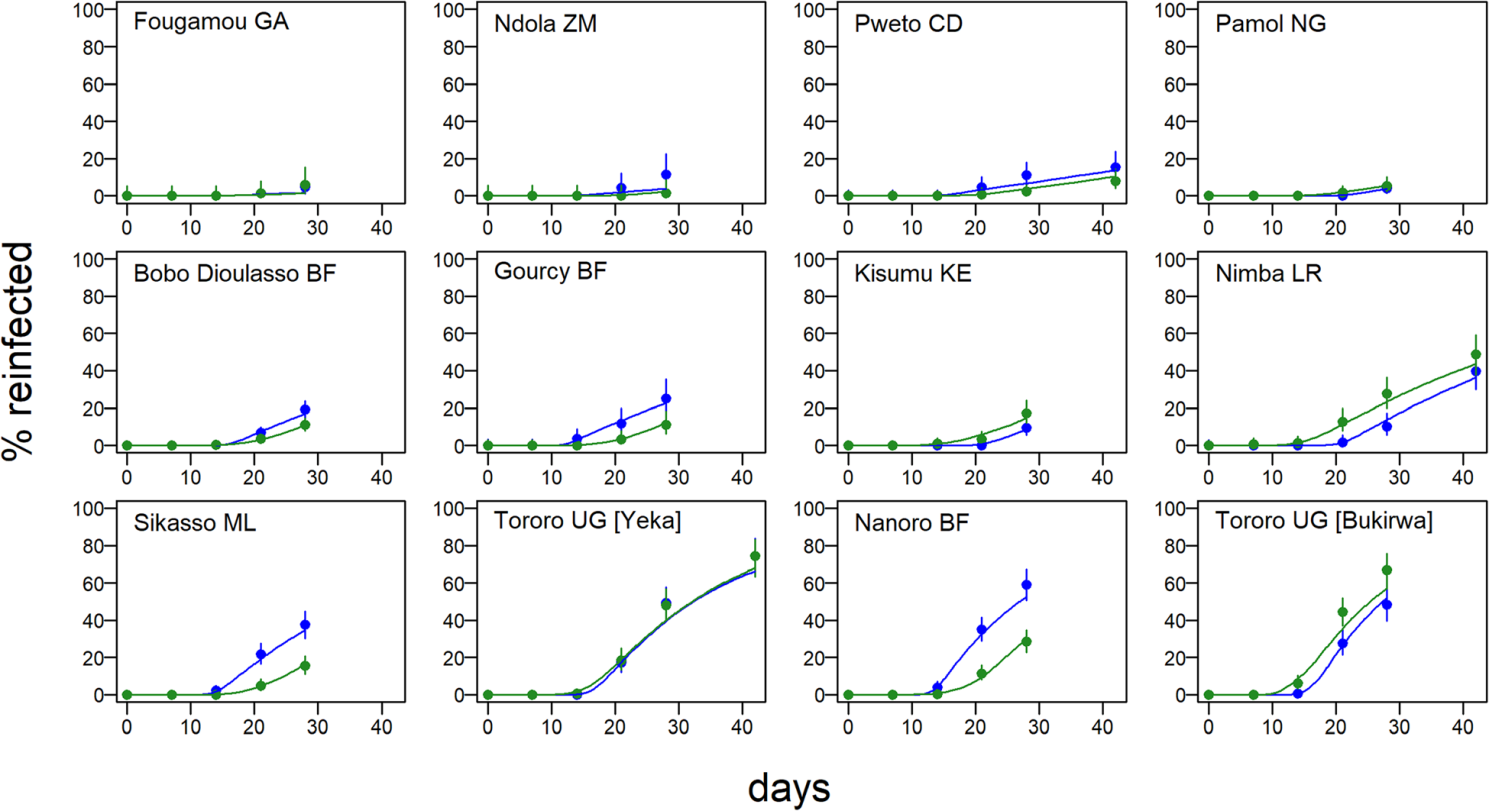
Time to reinfection after treatment and model fits. Proportion of patients reinfected (after PCR correction) during follow-up after treatment at day 0 with AL (blue) or AS-AQ (green) in each of the 12 trial sites. Circles show data with 95% CI, and the lines are the fits of the hidden semi-Markov model in each site. The AL trial arms include in total 2086 individuals and 642 reinfections and the AS-AQ trial arms, 2128 individuals and 538 reinfections

**Fig. 3:**
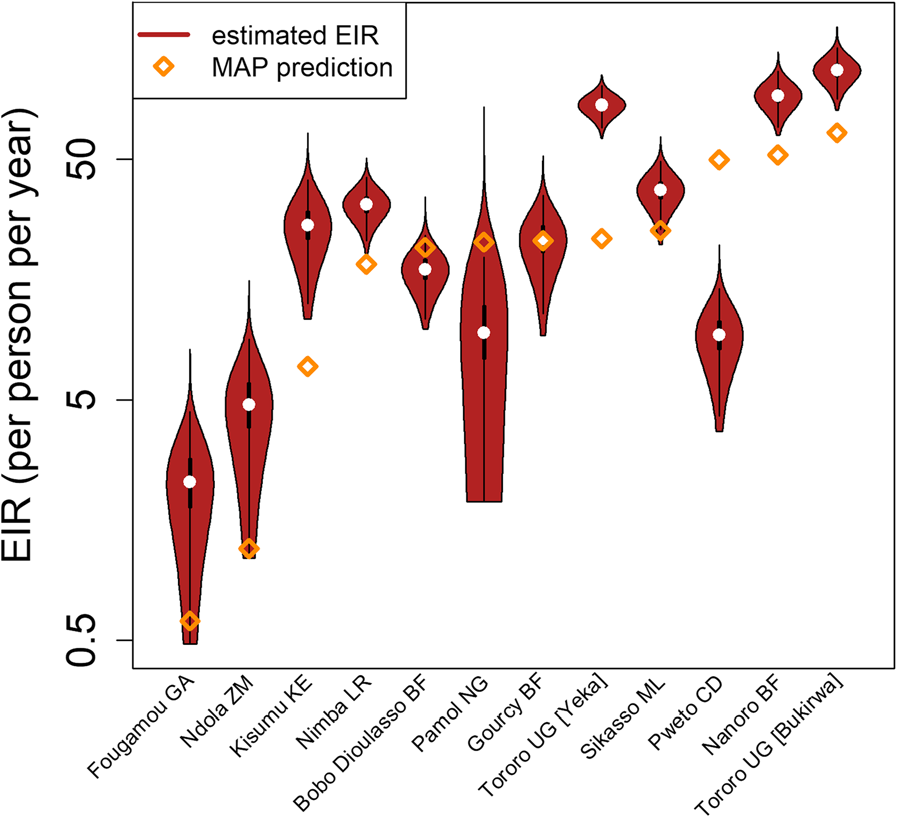
Trial-specific EIR estimates. Prior and posterior estimates of the EIR at each trial site. The prior predictions are based on Malaria Atlas Project data [28]

We analyzed 4214 individual participant data from randomized clinical trials in 12 sites. The median age in the study population was 2.8 years (IQR 1.5–4.2). With data pooled across trials, the median duration of protection against reinfection after AS-AQ treatment, i.e., the time during which patients have drug levels which would prevent reinfections, was estimated at 15.2 days (95% CI 12.8–18.4) and, after AL treatment, 13.0 days (95% CI 10.7–15.7) (Fig. 1). There appeared to be a more gradual transition from a protected to an unprotected state after treatment with AS-AQ compared to AL (Fig. 1b, c). However, the site-specific estimates of the duration of post-treatment prophylaxis for each drug were heterogeneous, with median estimates ranging from 10.2 to 18.7 days for AS-AQ and 8.7 to 18.6 days for AL (Fig. 1b, c, Table 1). The proportion of patients reinfected in the AS-AQ trial arm was lower than the AL arm in seven sites, while it was higher in the five other sites by the end of follow-up (Fig. 2). This heterogeneity was confirmed by the posterior estimates of the duration hyperparameters, which suggested non-zero variance of the random site effects. The heterogeneity existed despite the analysis taking into account variation in EIR, which ranged from an estimated 2 to 117 infectious bites per person per year, equating to an incidence of patent infection of 0.5–23.3 per person per year in this young study population. While there was, as expected, a reduced total time to reinfection with higher EIR, after accounting for EIR, we found no trend for duration of drug protection by EIR (Additional file 3: Figure S2). Overall, the model was able to fit the data well, with the model predicted values being within the 95% confidence intervals of the proportion of individuals reinfected at each follow-up time in almost all sites (Fig. 2). Posterior EIR values were mostly in line with the prior values but differed considerably for a small number of locations (Fig. 3, Table 1). For sensitivity analysis, we tried including additional age-independent variation in exposure to mosquito bites as in a previous analysis (see “Methods”), since this influences the distribution of reinfection times within a cohort. Such additional variation represents factors such as living close to a breeding site, housing quality, etc. This analysis found similar estimates of the duration of protection after AS-AQ and AL as did the model without additional variation in exposure, with medians of 16.5 days (95% CI 14.2–19.3) and 14.1 days (95% CI 11.7–16.9), respectively. Therefore, for parsimony, we did not include this factor in the final result. In a separate sensitivity analysis, carried out to allow for possible mistakes in PCR correction based on [65, 66], we reclassified a proportion of reinfections as recrudescences so that the total failure rate (% patients with recrudescence) in each trial arm increased by 3%. This caused only a slight increase in the estimated median duration of protection, to 15.6 days (95% CI 13.0–18.9) after AS-AQ and 13.8 days (95% CI 11.3–17.1) after AL (see also Additional file 4: Figure S3 for details).

### Factors affecting the duration of prophylaxis

To investigate which factors affect the duration of prophylaxis after AS-AQ and AL treatment and might explain the heterogeneity between trial sites, the data were further analyzed by accelerated failure-time regression models. As expected, estimated EIR was strongly associated with time to reinfection (Table 2). We therefore adjusted for EIR before testing the effect of any additional variables. Treatment arm had a small and significant effect on time to reinfection overall, with AS-AQ being associated with a 1.09-fold increase in time to reinfection (95% CI 1.05–1.13) compared to AL, after adjusting for log EIR. We explored the effect of molecular markers associated with parasite sensitivity to AL and AS-AQ, identifying *pfmdr1* 86Y surveys matching 11 trial sites, and *pfcrt* 76T matching 10 sites (matches are within 300 km of the trial site and within 1 year of the trial start or end year in the same country). However, there were too few matched surveys of *pfmdr1* 1246Y to analyze this third mutation further. Local prevalence of the mutations *pfmdr1* 86Y and *pfcrt* 76T significantly altered the association between drug and time to reinfection. AS-AQ was associated with a significant 1.37 (95% CI 1.28–1.47)-fold increase in time to reinfection compared to AL when *pfmdr1* 86Y prevalence was 20% (the lowest level observed in the trial sites), but a significantly shorter time to reinfection than AL when *pfmdr1* 86Y was 80% (ratio of reinfection times AS-AQ vs AL = 0.89 95% CI 0.84–0.94). Similarly, AS-AQ was associated with a 1.54 (95% CI 1.38–1.71)-fold increase in time to reinfection compared to AL when *pfcrt* 76T prevalence was 20%, but a 1.06 (95% CI 1.03–1.10)-fold change when *pfcrt* 76T prevalence was 80%. Other factors that were significantly associated with longer time to reinfection when adjusting each factor only for log EIR were younger age and higher dose of lumefantrine (mg per kg) (Table 2). Increasing age among children was associated with a shorter time to reinfection in a non-linear manner, such that the change in reinfection time with age was most rapid at younger ages, consistent with observed biting patterns by age [74]. There was a trend for shorter time to reinfection in underweight individuals and when the loose non-fixed-dose combination (NFDC) formulation of AS-AQ was used instead of the fixed-dose combination (FDC), though the association was not statistically significant after adjusting for log EIR.

**Table 2:** Risk factors for reinfection: analysis adjusted for EIR only. Data from 2130 individuals in the AS-AQ trial arms and 2090 in the AL trial arms were analyzed using accelerated failure-time analysis. Regression coefficients are the ratio of time to reinfection, such that a coefficient > 1 indicates a longer time to reinfection. All results are adjusted for log EIR. Site-level random effects were included unless otherwise indicated. Models assume a lognormal time to reinfection

Covariate (unit)	Analysis adjusted for EIR only		
	*N*	Coefficient [ratio of reinfection times] (95% CI)	*p* value
Log_e_ EIR (annual bites per person)	4220	0.79 (0.74, 0.85)	< 0.001
AL	2090	1 (ref)	
AS-AQ (overall)	2130	1.09 (1.05, 1.13)	< 0.001
AS-AQ (20% 86Y)*	1934	1.37 (1.28, 1.47)	< 0.001
AS-AQ (80% 86Y)*	1934	0.89 (0.84, 0.94)	< 0.001
Age (polynomial, years, > 20 grouped together)	4213		< 0.001
age		0.94 (0.90, 0.98)	
(age)^2^		1.01 (1.00, 1.02)	
(age)^3^		0.9998 (0.9994, 1.0001)	
Male gender	3861	0.98 (0.95, 1.02)	0.438
Anemic (hb < 10 g/dl)	3747	0.98 (0.93, 1.02)	0.277
Enlarged spleen^†^ (yes/no)	1390	1.00 (0.87, 1.15)	0.999
Presence of fever (> 37.5 °C)	4220	0.97 (0.93, 1.01)	0.146
Underweight (weight-for-age Z score < −2)	3193	0.98 (0.93, 1.04)	0.613
AQ dose (per 10 mg per kg increase) (AS-AQ arms only)	1839	1.00 (0.95, 1.06)	0.880
Lumefantrine dose (per 10 mg per kg increase) (AL arms only)	1850	1.02 (1.00, 1.04)	0.015
AS-AQ formulation			
FDC	1521	1 (ref)	
Loose NFDC	373	0.83 (0.59, 1.18)	0.295
Coblistered NFDC (AS-AQ arms only)	233	1.06 (0.68, 1.64)	0.803
*pfmdr1* 86Y prevalence (per 10% increase)			
AL arm^‡^	1891	1.03 (0.99, 1.07)	0.091
AS-AQ arm^‡^	1934	0.96 (0.94, 0.98)	< 0.001
*pfcrt* 76T prevalence (per 10% increase)			
AL arm^‡^	1964	1.03 (1.00, 1.07)	0.037
AS-AQ arm^‡^	2001	0.97 (0.95, 1.00)	0.052

^*^In a model including log_10_ EIR, drug, *pfmdr1* 86Y prevalence (per 10% increase) and interaction between drug and *pfmdr1* 86Y prevalence

^†^Site-level random effects not included because many sites did not measure this covariate

^‡^*p* value interaction between drug and *pfmdr1* 86Y vs N86 prevalence < 0.001, *p* value interaction between drug and *pfcrt* 76T vs K76 prevalence < 0.001

We constructed multivariable models for each treatment arm separately. In the AL arm, EIR, age, lumefantrine dose (mg per kg), local *pfmdr1* 86Y prevalence, and *pfcrt* 76T prevalence remained at least borderline significant predictors of time to reinfection (Table 3 and Additional file 1: Table S1). However, *pfmdr1* 86Y prevalence and *pfcrt* 76T prevalence were so closely correlated (Additional file 5: Figure S4) that their effects could not be distinguished from each other in the absence of haplotype data, and we built separate multivariable models to look at each mutation. In the AL arm, both the *pfmdr1* 86Y and the *pfcrt* 76T mutations were associated with a 1.04-fold increase in time to reinfection per 10% increase in their prevalence (*p* = 0.052 and *p* = 0.005, respectively) after adjusting for EIR, age, and lumefantrine dose.

**Table 3:** Risk factors for reinfection: multivariable analysis with *pfmdr1*. Data from 1934 individuals in the AS-AQ trial arms and 1655 in the AL trial arms were analyzed using accelerated failure-time analysis. Regression coefficients are the ratio of time to reinfection, such that a coefficient > 1 indicates a longer time to reinfection. Covariates significantly associated with reinfection time after adjusting for EIR (*p* < 0.05) were included in the final model. The prevalence of *pfcrt* 76T also had a significant effect in a multivariable model with the same covariates (Additional file 1: Table S1) but could not be included in the same model with *pfmdr1* 86Y due to strong correlation between the two variables. Models assume a lognormal time to reinfection and random site effects

Covariate (unit)	AL multivariable model (*N* = 1655) EIR, age, dose, *pfmdr1* 86Y		AS-AQ multivariable model (*N* = 1934) EIR, age, *pfmdr1* 86Y	
	Coefficient [ratio of reinfection times] (95% CI)	*p* value	Coefficient [ratio of reinfection times] (95% CI)	*p* value
Log_e_ annual EIR	0.81 (0.74, 0.90)	< 0.001	0.81 (0.75, 0.87)	< 0.001
Age (polynomial, years, > 20 grouped together)		< 0.001		< 0.001
age	1.01 (0.93, 1.09)		0.94 (0.88, 1.00)	
(age)^2^	1.00 (0.99, 1.02)		1.01 (1.00, 1.02)	
(age)^3^	1.0001 (0.9992, 1.0009)		0.9998 (0.9993, 1.0003)	
Lumefantrine dose (per 10 mg per kg increase) (AL arms only)	1.03 (1.01, 1.06)	0.002	–	–
*pfmdr1* 86Y prevalence (per 10% increase)	1.04 (1.00, 1.09)	0.059	0.97 (0.94, 0.99)	0.012

In the AS-AQ arm, EIR, age, and *pfmdr1* 86Y prevalence remained significantly associated with time to reinfection overall, with 86Y associated with a 0.97-fold decrease in reinfection time per 10% increase in prevalence (*p* = 0.011). For sensitivity analysis, we repeated the regression model including only the trial sites which used the FDC formulation of AS-AQ, and here the effect of *pfmdr1* 86Y was no longer statistically significant although the effect size remained similar (0.98 (95% CI 0.95, 1.01)-fold change in reinfection times, *p* = 0.159). Again, we looked at *pfcrt* 76T in a separate multivariable model in the AS-AQ arm; here, it was no longer significantly associated with reinfection time after adjusting for EIR and age, although there was still a trend for shorter time to reinfection as 76T prevalence increased (0.98-fold change in time to reinfection per 10% increase in 76T prevalence; 95% CI 0.95, 1.01).

**Fig. 4:**
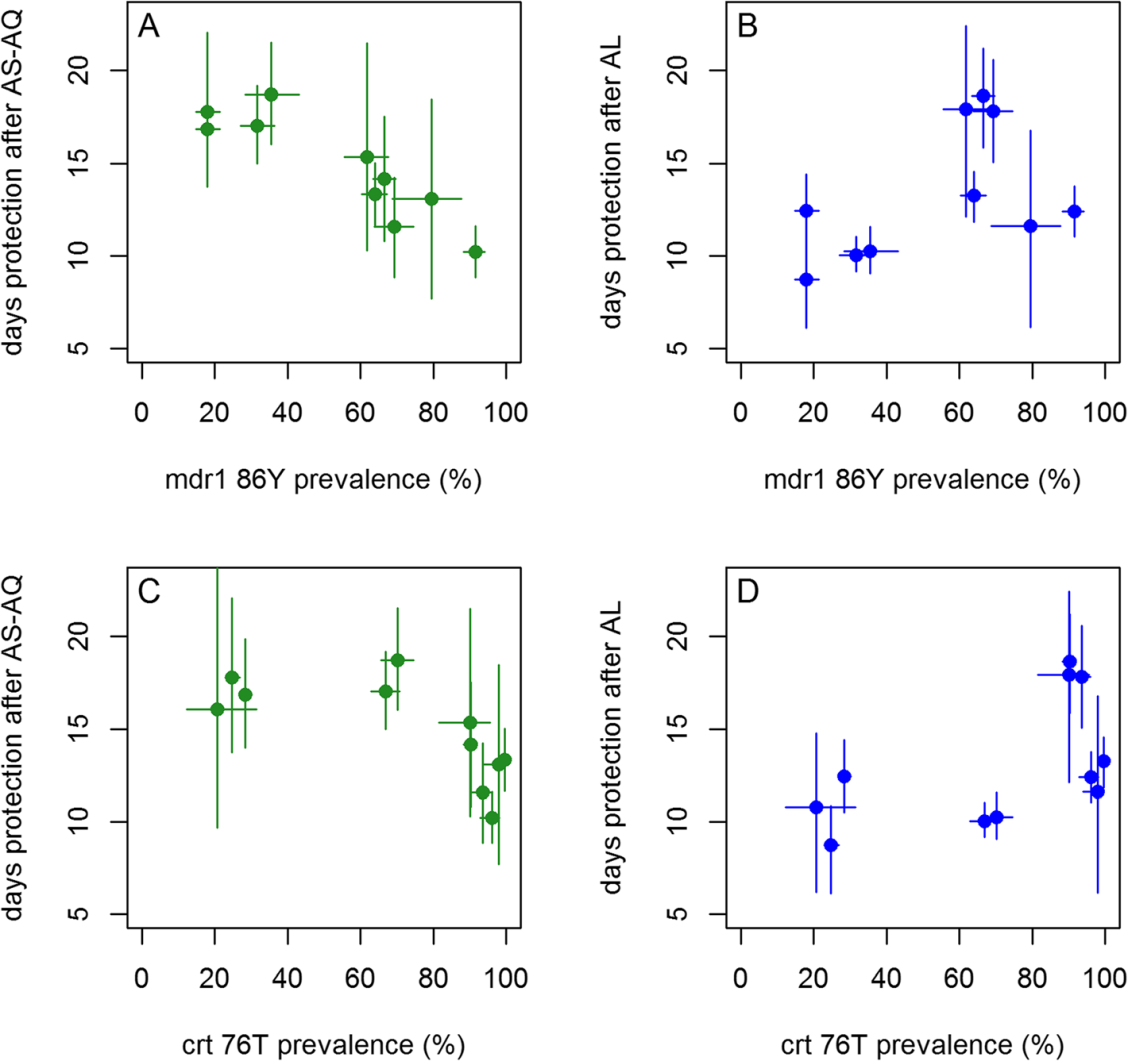
Duration of protection after treatment with **a**, **c** AS-AQ and **b**, **d** AL, according to local *pfmdr1* N86Y (**a**, **b**) and *pfcrt* K76T mutation prevalence (**c**, **d**). Median posterior estimates of duration of protection from hidden Markov model analysis are shown (points) with 95% credible intervals (vertical lines). Local *pfmdr1* N86Y and *pfcrt* K76T mutation prevalences are from matched surveys within 1 year and 300 km in the same country as each trial. Horizontal lines indicate the 95% confidence intervals of the mutation prevalence estimates

We further investigated the relationship of *pfmdr1* 86Y and *pfcrt* 76T prevalence with prophylactic time by examining the site-specific estimates from the hidden semi-Markov model analysis. The median estimated duration of protection (adjusted for EIR and age) was 16.9–17.8 days for AS-AQ in the trial sites with the lowest recorded 86Y and 76T prevalence (Bobo-Dioulasso and Gourcy in Burkina Faso), while it was 10.2–13.1 days in the trial sites with the highest 86Y and 76T prevalence (Tororo, Uganda and Fougamou, Gabon) (Fig. 4a, c). Conversely, the median duration of protection provided by AL was 8.7–12.5 days in the sites with the lowest 86Y and 76T prevalence, while in sites with higher 86Y and 76T prevalence, the duration of AL protection was variable but generally higher, at 11.5–18.6 days (Fig. 4b, d).

### Model-estimated population-level impact of using AS-AQ versus AL as first-line treatment

**Fig. 5:**
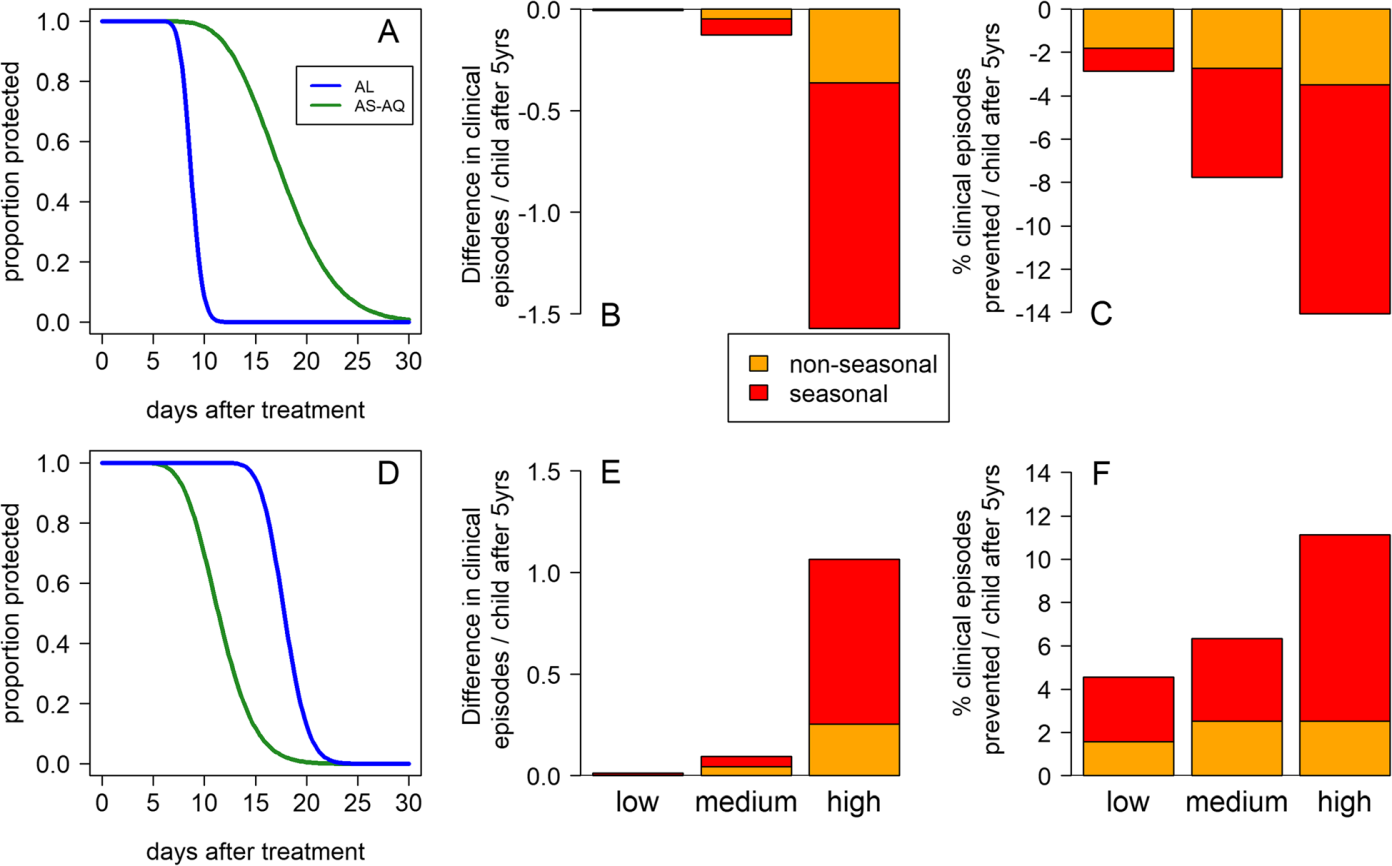
Duration of prophylaxis and impact on clinical incidence in under 5-year-old children of using AS-AQ rather than AL as first-line treatment, estimated by the transmission model analysis, contrasting areas with low (**a–c**) or high (**d–f**) *pfmdr1* 86Y and *pfcrt* 76T prevalence. **a** The estimated proportion of individuals protected over time since treatment by AL or AS-AQ in Gourcy, Burkina Faso, where 86Y and 76T prevalences are low (18% and 25%, respectively) and amodiaquine provides longer chemoprophylaxis than lumefantrine or **d** Nimba, Liberia, where 86Y and 76T prevalences are high (69% and 95%, respectively) and the prophylactic times are reversed so that lumefantrine provides longer chemoprophylaxis than amodiaquine. **b**, **c** The model-estimated impact in children aged 0–5 years of using AS-AQ rather than AL as first-line treatment in the whole population, using the prophylactic profiles in **a**. The outcomes are **b** the difference and **c** the % difference in the cumulative number of clinical episodes occurring during the 5 years after implementing either drug at 80% coverage; here AS-AQ is predicted to decrease clinical incidence compared with AL. Orange bars show the impact in non-seasonal settings, while red shows the impact in a seasonal setting (see “Methods”). **e**, **f** The corresponding results using the prophylactic profiles in **d**; here AS-AQ is predicted to increase clinical incidence compared with AL

The duration of prophylaxis provided by an antimalarial used as first-line treatment affects overall clinical incidence in a population because (a) it provides individual-level protection against reinfection and (b) prevention of reinfection reduces the total prevalence of infection in a population, and therefore onward transmission from infected individuals. Simulations comparing the public health impact of using either AL or AS-AQ as first-line drug were run using the existing individual-based age-structured mathematical model of *Plasmodium falciparum* transmission. Given the variation in prophylactic time between areas, we chose to use estimates from two of the trial sites with the most contrasting effects of the two drugs (Fig. 5). In the trial in Gourcy, Burkina Faso in 2010–2012, there was low local prevalence of the *pfmdr1* 86Y mutation (18%) and the *pfcrt* 76T mutation (25%), with a correspondingly long estimated median duration of protection by AS-AQ at 17.8 days, approximately twice as long as the median duration of protection by AL in this site: 8.7 days. Using the prophylactic profiles estimated in this trial site (Fig. 5a), we introduced either AL or AS-AQ as first-line treatment into our simulation, assuming 80% of clinical episodes in all ages are treated with this drug, and the total number of clinical episodes occurring in 0–5-year-olds over the subsequent 5 years was compared between the two treatments. The longer prophylactic time of AS-AQ reduced clinical episodes in all transmission scenarios (Fig. 5b, c), but was most pronounced in simulations with higher, very seasonal transmission. When slide prevalence was 50% and transmission was seasonal, using AS-AQ rather than AL prevented 1.6 clinical episodes per child over the 5 years (Fig. 5b) (14% of all clinical episodes; Fig. 5c). When considering all age groups, an estimated 10% of clinical episodes were prevented (Additional file 6: Figure S5).

In Nimba in Liberia (trial conducted 2008–2009), the local prevalence of *pfmdr1* 86Y and *pfcrt 76T* were much higher at 69% and 95%, and the median duration of prophylaxis provided by AS-AQ was estimated at only 11.6 days, while the median AL prophylactic time was 17.9 days (Fig. 5d). Here, using AS-AQ rather than AL increased the cumulative number of clinical episodes per 0–5-year-old child by up to 1.1 over the 5-year simulated period (an increase of 11%), with the largest difference between drugs again observed in the very seasonal, high transmission scenario (Fig. 5e, f). When considering all age groups, clinical episodes increased by up to 8% (Additional file 6: Figure S5).

In both settings, there was minimal difference in impact on clinical episodes (< 1%) if we assumed that patients treated with AL were half as infectious as those treated with AS-AQ, compared with the scenarios where infectiousness was assumed to be equal after each treatment (results not shown). This is because even if there is some difference between treatments, both are estimated to have a high impact on gametocytes. Therefore, at a population level, transmission to mosquitoes is dominated by untreated infections which are thought to last on average about 6 months, according to our model assumptions and parameters [69, 77, 78].

## Discussion

In this analysis of clinical trials from 12 sites in Africa, we initially estimated that AS-AQ provided a slightly longer median duration of post-treatment prophylaxis than AL (15.2 versus 13.0 days) when all data were pooled together. However, the duration of protection varied considerably between trial sites. In some locations, AS-AQ provided up to an estimated 19 days of protection, ~ 2-fold longer than AL, while in other trial sites the reverse was true, with AL providing up to 19 days of protection, which was up to 1.5-fold longer than AS-AQ. This difference between sites appeared to be in part explained by the local prevalence of *pfmdr1* 86Y and *pfcrt* 76T at the time of the trial, with AS-AQ providing better protection where wild type parasites with N86 and K76 genotypes were predominant, and AL performing better where 86Y and 76T mutants were common. This is consistent with previous studies demonstrating the collateral sensitivity of parasites with these different *pfmdr1* and *pfcrt* genotypes to AL and AS-AQ. Our analysis extends previous work [9, 11, 79] by explicitly estimating the duration of protection provided by each drug in sites with different prevalence of 86Y and 76T mutants, also taking into account the different EIRs across the trial sites so as to distinguish the effect of the drugs from that of the local transmission intensity on the time to reinfection.

Our transmission modeling suggests that the difference in duration of protection between the two drugs in areas with very low or very high *mdr1* 86Y and *crt* 76T prevalence can have a public health impact, especially where malaria transmission is high and seasonal. We estimate that up to 14% of clinical episodes could be prevented in 0–5-year-old children by implementing first-line treatment with the drug providing optimal protection in a given setting, due to both individual protection from reinfection and population-level reduction in transmission (when 80% of clinical episodes receive treatment). Countries with low (< 20%) or high (> 80%) prevalence of 86Y and 76T and intense transmission could consider the benefit of longer duration of protection if choosing between AL and AS-AQ policies. Using a first-line treatment with longer duration of protection is potentially a cost-effective way of reducing clinical cases and infections [4] given the comparable price of AL and AS-AQ [80]. Compared to published estimates, both AL and AS-AQ provided a shorter duration of protection than dihydroartemisinin-piperaquine (estimated at 29.4 days of > 50% protection [4]), which is predicted to prevent up to 15% more cases than AL [4, 81].

The *pfmdr1* 86Y and *pfcrt* 76T mutations, initially driven through the parasite population by the previous widespread use of chloroquine, have been in decline in many parts of Africa. The decline has occurred fastest in countries using AL, consistent with the expected direction of selection [68]. The efficacy of AS-AQ appears to have improved in some countries and there is interest in increasing the use of the drug regimen [55]. Our results suggest that some countries with areas of high transmission who currently use AL might gain better post-treatment protection by deploying AS-AQ (e.g., Uganda [79], southern Tanzania, western Kenya) if feasible given other considerations (logistics of changing drug policy, adherence, acceptability etc.). The prevalence of mutations or the prophylactic benefits may need to be monitored. Amodiaquine is also widely used together with SP in seasonal malaria chemoprevention (SMC) programs in children in the Sahel region of Africa, given to 17 million children under 5 years of age in 2016 [7, 82]. Our results could be used together with information on the chemoprophylaxis provided by SP, to inform potential changes in the efficacy of SMC as 86Y and 76T prevalence change. The decline in 86Y in many areas may have enhanced the efficacy of SP-AQ. This may be particularly important in areas with partial SP resistance. Our results support previous findings suggesting that selective pressures exerted by AL and AS-AQ may counteract each other. However, our results suggest it would not be possible to achieve maximal prophylactic effect of either AL or AS-AQ at the same time in a given setting. Triple ACT which combine an artemisinin derivative with both lumefantrine and amodiaquine are currently in trials [83] and would be likely to ensure longer prophylactic protection.

Our finding that the *pfmdr1* 86Y and *pfcrt* 76T mutations are associated with a longer time to reinfection after AL treatment and a shorter time after AS-AQ is consistent with a previous meta-analysis, where individual patient data on genotypes post-treatment were available [9, 11]. We did not include such a wide range of studies as the previous meta-analysis because our methods required that we estimate the EIR for each included trial site, which is only possible when sufficient numbers of reinfections are observed per site and we included only randomized trials. The advantage of our approach, however, is that we can obtain estimates of prophylactic times after adjusting for the local transmission intensity. One limitation of our study was that we did not have individual-level data on genotypes pre and post-treatment, which were not measured in the trials we included here. This might have allowed a more precise estimate of the effect of mutations on prophylactic time and ideally comparison of different *pfcrt* and *pfmdr1* haplotypes. Also, while we matched trials to the closest possible measures of mutation prevalence, these may not reflect the prevalence in the trial sites which can vary over space and time. We could not distinguish separate effects of 86Y and 76T in this analysis due to the close correlation of their prevalence. Other previous meta-analyses have examined the effect of dosing and other covariates on the probability of recrudescence after AL [10] and AS-AQ [12]. The trends in our analysis looking at reinfection as the outcome rather than recrudescence agree well with these previous studies; in particular, the use of loose NFDC formulation of AS-AQ was associated with reduced time to reinfection although it was not statistically significant after adjusting for EIR. Of the three studies using loose NFDC, two of these showed a longer prophylactic time by AL, compared to two out of the remaining 9 studies which used FDC.

Our estimate of the mean duration of prophylaxis after AL at 13.0 days is in good agreement with our previous estimate of 13.8 days which was obtained from analysis of a completely different dataset of clinical trials in six sites in Africa [4] (although the impact of 86Y and 76T was not previously investigated). Our estimates of duration of prophylaxis for both drugs are affected by the assumed time from release of parasites from the liver until they multiply to densities detectable by microscopy. We assumed this time to patency is 3.5 days, but estimates vary from about 2–7 days depending on several factors, including the assumed number of parasites released from hepatocytes at the start of blood-stage infection (~ 100,000–300,000 [84]), the volume of blood in an individual (relatively low in the young children in the included trials), and the sensitivity of microscopy. The time to patency is further complicated by the presence of residual drug concentrations which might slow parasite growth. A longer time to patency would reduce our estimate of the duration of protection. Our estimates of duration of prophylaxis are also dependent to some extent on the priors used for estimating EIR in each site, without which we cannot distinguish between low infection rates and long duration of prophylaxis. The agreement of our estimate of prophylaxis for AL with our previous estimate from different trial sites with different EIR, together with the biologically plausible association of duration of prophylaxis with *mdr1* and *crt* mutation prevalence, is reassuring.

In the current analysis, we found a more rapid decline of protection over time after AL treatment than AS-AQ (Fig. 1), and a similar rapid decline after AL was seen in our previous analysis. The resolution of data informing this profile of post-treatment prophylaxis is not perfect, with most patients observed only weekly after day 7. In 4 of the trial sites in the current analysis, no tests for reinfection were done until day 14 [23]. Nevertheless, given the very low proportion of individuals reinfected at earlier times in the other sites, it is unlikely that many reinfections were missed. In most trials, the patients were followed up until day 28, and differential reinfection rates may have been missed after this time. We lacked data from a control arm to parameterize the proportion of individuals reinfected over time in the absence of treatment. If our model underestimates the rate of increase in the proportion of individuals reinfected in the absence of treatment, it could overestimate the rapid drop off in protection in the AL trial arms to compensate. There is therefore some uncertainty in the shape of the prophylactic profile but if the rapid drop in protection is a real finding, it has implications for the selection of partially resistant parasites to these partner drugs, with lumefantrine potentially having a relatively short window of selection compared to amodiaquine [85].

We also did not consider temporal changes in the EIR during the trial. However, these would affect both trial arms equally and could therefore not reverse the relative order of duration of protection between the drugs in one site. Variation between studies may occur due to other factors such as nutritional status, dosage, the genetics of patients, or variations in the accuracy of PCR in distinguishing reinfections from recrudescence. While none of the trials distributed insecticide-treated nets as part of the study, trial areas probably varied in levels of vector control, which is indirectly taken into account in our analysis since we use estimates of transmission intensity based on the Malaria Atlas Project, who use data on prevalence trends and include vector control in their model.

## Conclusions

In summary, both AL and AS-AQ provide post-treatment prophylaxis which is important for reducing reinfection rates in individuals in higher transmission settings and may impact on the incidence of malaria in the whole population when these regimens are used widely as first-line treatment. AS-AQ provides longer protection than AL when most infections are by wild type parasites, while AL provides longer protection than AS-AQ in areas with higher prevalence of the *pfmdr1* 86Y and *pfcrt* 76T mutations. Countries may wish to consider the prevalence of these mutations when deciding the first-line treatment. In future, it will be important to determine the role of other molecular markers in altering the post-treatment protection provided by ACT partner drugs, such as increased copy number of *pfmdr1*, which is increasing in prevalence in some parts of Africa [67].

## Supplementary information

**Additional file 1: Table S1.** Risk factors for reinfection: multivariable analysis with *pfcrt* 76T. Data from AS-AQ and AL trial arms were analyzed separately using accelerated failure-time analysis. Regression coefficients are the ratio of time to reinfection, such that a coefficient > 1 indicates a longer time to reinfection. Covariates significantly associated with reinfection time after adjusting for EIR (Table 3, main text) were included in the final model. The prevalence of *pfmdr1* 86Y also had a significant effect in a multivariable model with the same covariates (Table 3, main text) but could not be included in the same model with *pfcrt* 76T due to strong correlation between the two variables. Models assume a log-normal time to reinfection and random site effects.

**Additional file 2:Figure S1.** Simulated annual seasonal variation in EIR assumed in the analysis of potential impact of AL and AS-AQ on population level transmission (Fig. 5, main text). The EIR shown is for the simulated seasonal medium transmission setting (slide prevalence = 15%), but the relative EIR variation across the year was the same in the seasonal low and high simulated transmission settings.

**Additional file 3: Figure S2.** The duration of post-treatment prophylaxis at different trial locations in order of increasing estimated EIR. Posterior estimates of the duration of protection provided by AL or AS-AQ are shown. The study sites are shown in order of increasing transmission intensity left to right according to posterior EIR estimates.

**Additional file 4: Figure S3.** Sensitivity analysis of PCR-correction misclassification: time to reinfection after treatment and model fits. Here we repeated the analysis shown in Fig. 2 of the main text on a modified dataset, in which we explored the impact of reclassifying some reinfections as recrudescences and removing them from the analysis. To reclassify reinfections for each trial arm, we sampled a number of reinfections equal to 3% of the study population, with probability weighted according to the estimated timings of recrudescence in Fig. 5 of [66] and the relative frequency of apparent ‘reinfection’ timing in the current dataset (such that the probabilities of sampling of reinfections, if present, at days 7,14,21,28,35, and 42 were 0.799, 0.100, 0.026, 0.014, 0.049, and 0.012). One site, Ndola in Zambia, was excluded after reclassification of reinfections, since one trial arm no longer contained reinfections and the model could not be fitted. The Figure shows the proportion of patients reinfected during follow up, amongst patients not experiencing recrudescence, after treatment at day 0 with AL (blue) or AS-AQ (green) in each of the 11 trial sites included in this sensitivity analysis. Circles show data with 95% CI, and the lines are the fits of the hidden semi-Markov model in each site. Here, the AL trial arms include in total 1956 individuals, 573 reinfections, and the AS-AQ trial arms, 2001 individuals, 475 reinfections.

**Additional file 5: Figure S4.** Correlation between *pfcrt* 76T prevalence and *pfmdr* 86Y prevalence, in the surveys matched to the trial sites according to year and geographic distance (within 1 year and 300 km in the same country as each trial). When more than one molecular marker survey was matched to a trial site, a weighted average prevalence was taken. In some cases, these two molecular markers were assessed in the same matched survey(s), but in other cases matches from different surveys were found.

**Additional file 6: Figure S5.** As Fig. 5 in the main text, except panels B,C,E and F show impact on clinical incidence in the whole population (rather than 0–5 year old children only).

## Data Availability

Analysis code in R and the transmission model executable file are fully available online at https://github.com/lucyokell/duration_protection_AL_ASAQ, as are the data underlying the figures: (Zenodo data repository DOI 10.5281/zenodo.3339215). The source code for the transmission model in C++ is available on Github https://github.com/jamiegriffin/Malaria_simulation. The original individual-level clinical trial data is available upon request from WWARN (https://www.wwarn.org/accessing-data). Requests must be approved by the data contributor and the WWARN Malaria Data Access Committee.

## References

[1] World Health Organization (2015). WHO Guidelines for the treatment of malaria.

[2] World Health Organization (2018). Q&A on artemisinin resistance.

[3] malERA (2011). A research agenda for malaria eradication: drugs. PLoS Med.

[4] Okell LC, Cairns M, Griffin JT, Ferguson NM, Tarning J, Jagoe G (2014). Contrasting benefits of different artemisinin combination therapies as first-line malaria treatments using model-based cost-effectiveness analysis. Nat Commun.

[5] Okell LC, Drakeley CJ, Bousema T, Whitty CJ, Ghani AC (2008). Modelling the impact of artemisinin combination therapy and long-acting treatments on malaria transmission intensity. PLoS Med.

[6] Cairns M, Ghani A, Okell L, Gosling R, Carneiro I, Anto F (2011). Modelling the protective efficacy of alternative delivery schedules for intermittent preventive treatment of malaria in infants and children. PLoS One.

[7] World Health Organization. World Malaria Report 2018. Accessed at https://www.who.int/malaria/publications/world-malaria-report-2018/en/ 05 Jan 2019.

[8] Tun KM, Imwong M, Lwin KM, Win AA, Hlaing TM, Hlaing T (2015). Spread of artemisinin-resistant Plasmodium falciparum in Myanmar: a cross-sectional survey of the K13 molecular marker. Lancet Infect Dis.

[9] Venkatesan M, Gadalla NB, Stepniewska K, Dahal P, Nsanzabana C, Moriera C, et al. Polymorphisms in *Plasmodium falciparum* chloroquine resistance transporter and multidrug resistance 1 genes: parasite risk factors that affect treatment outcomes for *P. falciparum* malaria after artemether-lumefantrine and artesunate-amodiaquine. Am J Trop Med Hyg Erratum https://www.wwarnorg/sites/default/files/attachments/documents/erratum-full-paper-polymorphisms-pfcrt-pfmdr1-ajtmh-november-2019pdf. 2014 Erratum 2019;91(4):833–43. 10.4269/ajtmh.14-0031 PMC4183414 25048375

[10] Worldwide Antimalarial Resistance Network (WWARN) AL Dose Impact Study Group (2015). The effect of dose on the antimalarial efficacy of artemether-lumefantrine: a systematic review and pooled analysis of individual patient data. Lancet Infect Dis.

[11] Erratum for Polymorphisms in Plasmodium falciparum chloroquine resistance transporter and multidrug resistance 1 genes: parasite risk factors that affect treatment outcomes for P. falciparum malaria after artemether-lumefantrine and artesunate-amodiaquine. [Am J Trop Med Hyg. 2014]. Erratum Am J Trop Med Hyg. 2019;100(3):766. 10.4269/ajtmh.14-0031 PMC4183414 25048375

[12] Worldwide Antimalarial Resistance Network (WWARN) AS-AQ Study Group (2015). The effect of dosing strategies on the therapeutic efficacy of artesunate-amodiaquine for uncomplicated malaria: a meta-analysis of individual patient data. BMC Med.

[13] Ashley EA, Stepniewska K, Lindegardh N, McGready R, Annerberg A, Hutagalung R (2007). Pharmacokinetic study of artemether-lumefantrine given once daily for the treatment of uncomplicated multidrug-resistant falciparum malaria. Tropical Med Int Health.

[14] Tarning J, Kloprogge F, Dhorda M, Jullien V, Nosten F, White NJ (2013). Pharmacokinetic properties of artemether, dihydroartemisinin, lumefantrine, and quinine in pregnant women with uncomplicated plasmodium falciparum malaria in Uganda. Antimicrob Agents Chemother.

[15] Djimde A, Lefevre G (2009). Understanding the pharmacokinetics of Coartem. Malar J.

[16] Kloprogge F, McGready R, Hanpithakpong W, Blessborn D, Day NP, White NJ (2015). Lumefantrine and desbutyl-lumefantrine population pharmacokinetic-pharmacodynamic relationships in pregnant women with uncomplicated plasmodium falciparum malaria on the Thailand-Myanmar border. Antimicrob Agents Chemother.

[17] Tarning J, Chotsiri P, Jullien V, Rijken MJ, Bergstrand M, Cammas M (2012). Population pharmacokinetic and pharmacodynamic modeling of amodiaquine and desethylamodiaquine in women with plasmodium vivax malaria during and after pregnancy. Antimicrob Agents Chemother.

[18] Adjei GO, Kristensen K, Goka BQ, Hoegberg LC, Alifrangis M, Rodrigues OP (2008). Effect of concomitant artesunate administration and cytochrome P4502C8 polymorphisms on the pharmacokinetics of amodiaquine in Ghanaian children with uncomplicated malaria. Antimicrob Agents Chemother.

[19] Hietala SF, Bhattarai A, Msellem M, Roshammar D, Ali AS, Stromberg J (2007). Population pharmacokinetics of amodiaquine and desethylamodiaquine in pediatric patients with uncomplicated falciparum malaria. J Pharmacokinet Pharmacodyn.

[20] Hombhanje FW, Hwaihwanje I, Tsukahara T, Saruwatari J, Nakagawa M, Osawa H (2005). The disposition of oral amodiaquine in Papua new Guinean children with falciparum malaria. Brit J Clin Pharmaco.

[21] Mwesigwa J, Parikh S, McGee B, German P, Drysdale T, Kalyango JN (2010). Pharmacokinetics of artemether-lumefantrine and artesunate-amodiaquine in children in Kampala, Uganda. Antimicrob Agents Chemother.

[22] Stepniewska K, Taylor W, Sirima SB, Ouedraogo EB, Ouedraogo A, Gansane A (2009). Population pharmacokinetics of artesunate and amodiaquine in African children. Malar J.

[23] 4ABC Study Group (2011). A head-to-head comparison of four artemisinin-based combinations for treating uncomplicated malaria in African children: a randomized trial. PLoS Med.

[24] Gosling RD, Cairns ME, Chico RM, Chandramohan D (2010). Intermittent preventive treatment against malaria: an update. Expert Rev Anti-Infect Ther.

[25] Worldwide antimalarial resistance network (WWARN). http://www.wwarn.org/.

[26] WorldWide Antimalarial Resistance Network AS-AQ Post-Treatment Prophylaxis Study Group. http://www.wwarn.org/working-together/study-groups/aq-post-treatment-prophylaxis-study-group. Accessed 2 Mar 2019.

[27] WWARN (2012). Clinical Module: Data Management and Statistical Analysis Plan. Version 1.2.

[28] Malaria Atlas Project. 2015. Accessed at http://www.map.ox.ac.uk/data/ 07 July 2016.

[29] Bhatt S, Weiss DJ, Cameron E, Bisanzio D, Mappin B, Dalrymple U (2015). The effect of malaria control on Plasmodium falciparum in Africa between 2000 and 2015. Nature..

[30] Mawili-Mboumba DP, Ndong Ngomo JM, Maboko F, Guiyedi V, Mourou Mbina JR, Kombila M (2014). Pfcrt 76T and pfmdr1 86Y allele frequency in Plasmodium falciparum isolates and use of self-medication in a rural area of Gabon. Trans R Soc Trop Med Hyg.

[31] Frank M, Lehners N, Mayengue PI, Gabor J, Dal-Bianco M, Kombila DU (2011). A thirteen-year analysis of Plasmodium falciparum populations reveals high conservation of the mutant pfcrt haplotype despite the withdrawal of chloroquine from national treatment guidelines in Gabon. Malar J.

[32] Sagara I, Oduro AR, Mulenga M, Dieng Y, Ogutu B, Tiono AB (2014). Efficacy and safety of a combination of azithromycin and chloroquine for the treatment of uncomplicated plasmodium falciparum malaria in two multi-country randomised clinical trials in African adults. Malar J.

[33] Espie E, Lima A, Atua B, Dhorda M, Flevaud L, Sompwe EM (2012). Efficacy of fixed-dose combination artesunate-amodiaquine versus artemether-lumefantrine for uncomplicated childhood Plasmodium falciparum malaria in Democratic Republic of Congo: a randomized non-inferiority trial. Malar J.

[34] Happi CT, Gbotosho GO, Folarin OA, Sowunmi A, Hudson T, O'Neil M (2009). Selection of Plasmodium falciparum multidrug resistance gene 1 alleles in asexual stages and gametocytes by artemether-lumefantrine in Nigerian children with uncomplicated falciparum malaria. Antimicrob Agents Chemother.

[35] Folarin OA, Bustamante C, Gbotosho GO, Sowunmi A, Zalis MG, Oduola AM (2011). In vitro amodiaquine resistance and its association with mutations in pfcrt and pfmdr1 genes of Plasmodium falciparum isolates from Nigeria. Acta Trop.

[36] Oladipo OO, Wellington OA, Sutherland CJ (2015). Persistence of chloroquine-resistant haplotypes of Plasmodium falciparum in children with uncomplicated malaria in Lagos, Nigeria, four years after change of chloroquine as first-line antimalarial medicine. Diagn Pathol.

[37] Ojurongbe O, Oyedeji SI, Oyibo WA, Fagbenro-Beyioku AF, Kun JF (2010). Molecular surveillance of drug-resistant Plasmodium falciparum in two distinct geographical areas of Nigeria. Wien Klin Wochenschr.

[38] Some AF, Zongo I, Compaore YD, Sakande S, Nosten F, Ouedraogo JB (2014). Selection of drug resistance-mediating Plasmodium falciparum genetic polymorphisms by seasonal malaria chemoprevention in Burkina Faso. Antimicrob Agents Chemother.

[39] Sondo P, Derra K, Diallo Nakanabo S, Tarnagda Z, Kazienga A, Zampa O (2016). Artesunate-amodiaquine and artemether-lumefantrine therapies and selection of Pfcrt and Pfmdr1 alleles in Nanoro, Burkina Faso. PLoS One.

[40] Some AF, Sorgho H, Zongo I, Bazie T, Nikiema F, Sawadogo A (2016). Polymorphisms in K13, pfcrt, pfmdr1, pfdhfr, and pfdhps in parasites isolated from symptomatic malaria patients in Burkina Faso. Parasite..

[41] Sondo P, Derra K, Tarnagda Z, Nakanabo SD, Zampa O, Kazienga A (2015). Dynamic of plasmodium falciparum chloroquine resistance transporter gene Pfcrt K76T mutation five years after withdrawal of chloroquine in Burkina Faso. Pan Afr Med J.

[42] Zongo I, Milligan P, Compaore YD, Some AF, Greenwood B, Tarning J (2015). Randomized noninferiority trial of dihydroartemisinin-piperaquine compared with sulfadoxine-pyrimethamine plus amodiaquine for seasonal malaria chemoprevention in Burkina Faso. Antimicrob Agents Chemother.

[43] Holmgren G, Bjorkman A, Gil JP (2006). Amodiaquine resistance is not related to rare findings of pfmdr1 gene amplifications in Kenya. Tropical Med Int Health.

[44] Zhong D, Afrane Y, Githeko A, Cui L, Menge DM, Yan G (2008). Molecular epidemiology of drug-resistant malaria in western Kenya highlands. BMC Infect Dis.

[45] Vardo-Zalik AM, Zhou G, Zhong D, Afrane YA, Githeko AK, Yan G (2013). Alterations in Plasmodium falciparum genetic structure two years after increased malaria control efforts in western Kenya. Am J Trop Med Hyg.

[46] Bonizzoni M, Afrane Y, Baliraine FN, Amenya DA, Githeko AK, Yan G (2009). Genetic structure of plasmodium falciparum populations between lowland and highland sites and antimalarial drug resistance in Western Kenya. Infect Genet Evol.

[47] Spalding MD, Eyase FL, Akala HM, Bedno SA, Prigge ST, Coldren RL (2010). Increased prevalence of the pfdhfr/phdhps quintuple mutant and rapid emergence of pfdhps resistance mutations at codons 581 and 613 in Kisumu, Kenya. Malar J.

[48] Schramm B, Valeh P, Baudin E, Mazinda CS, Smith R, Pinoges L (2013). Efficacy of artesunate-amodiaquine and artemether-lumefantrine fixed-dose combinations for the treatment of uncomplicated Plasmodium falciparum malaria among children aged six to 59 months in Nimba County, Liberia: an open-label randomized non-inferiority trial. Malar J.

[49] Otienoburu SD, Maiga-Ascofare O, Schramm B, Jullien V, Jones JJ, Zolia YM (2016). Selection of Plasmodium falciparum pfcrt and pfmdr1 polymorphisms after treatment with artesunate-amodiaquine fixed dose combination or artemether-lumefantrine in Liberia. Malar J.

[50] Sagara I, Fofana B, Gaudart J, Sidibe B, Togo A, Toure S (2012). Repeated artemisinin-based combination therapies in a malaria hyperendemic area of Mali: efficacy, safety, and public health impact. Am J Trop Med Hyg.

[51] Tekete M, Djimde AA, Beavogui AH, Maiga H, Sagara I, Fofana B (2009). Efficacy of chloroquine, amodiaquine and sulphadoxine-pyrimethamine for the treatment of uncomplicated falciparum malaria: revisiting molecular markers in an area of emerging AQ and SP resistance in Mali. Malar J.

[52] Andriantsoanirina V, Menard D, Rabearimanana S, Hubert V, Bouchier C, Tichit M (2010). Association of microsatellite variations of Plasmodium falciparum Na+/H+ exchanger (Pfnhe-1) gene with reduced in vitro susceptibility to quinine: lack of confirmation in clinical isolates from Africa. Am J Trop Med Hyg.

[53] Djimde AA, Fofana B, Sagara I, Sidibe B, Toure S, Dembele D (2008). Efficacy, safety, and selection of molecular markers of drug resistance by two ACTs in Mali. Am J Trop Med Hyg.

[54] Doumbo S, Ongoiba OA, Doumtabe D, Dara A, Ouologuem TD, Kayentao K (2013). Prevalence of Plasmodium falciparum, anemia and molecular markers of chloroquine and sulfadoxine-pyrimethamine resistance in delivered women in Fana, Mali. Bull Soc Pathol Exot.

[55] Yeka A, Lameyre V, Afizi K, Fredrick M, Lukwago R, Kamya MR (2014). Efficacy and safety of fixed-dose artesunate-amodiaquine vs. artemether-lumefantrine for repeated treatment of uncomplicated malaria in Ugandan children. PLoS One.

[56] Mbogo GW, Nankoberanyi S, Tukwasibwe S, Baliraine FN, Nsobya SL, Conrad MD (2014). Temporal changes in prevalence of molecular markers mediating antimalarial drug resistance in a high malaria transmission setting in Uganda. Am J Trop Med Hyg.

[57] Tumwebaze P, Conrad MD, Walakira A, LeClair N, Byaruhanga O, Nakazibwe C (2015). Impact of antimalarial treatment and chemoprevention on the drug sensitivity of malaria parasites isolated from ugandan children. Antimicrob Agents Chemother.

[58] Tukwasibwe S, Mugenyi L, Mbogo GW, Nankoberanyi S, Maiteki-Sebuguzi C, Joloba ML (2014). Differential prevalence of transporter polymorphisms in symptomatic and asymptomatic falciparum malaria infections in Uganda. J Infect Dis.

[59] Some AF, Sere YY, Dokomajilar C, Zongo I, Rouamba N, Greenhouse B (2010). Selection of known plasmodium falciparum resistance-mediating polymorphisms by artemether-lumefantrine and amodiaquine-sulfadoxine-pyrimethamine but not dihydroartemisinin-piperaquine in Burkina Faso. Antimicrob Agents Chemother.

[60] Bukirwa H, Yeka A, Kamya MR, Talisuna A, Banek K, Bakyaita N (2006). Artemisinin combination therapies for treatment of uncomplicated malaria in Uganda. PLoS Clin Trials.

[61] Dokomajilar C, Nsobya SL, Greenhouse B, Rosenthal PJ, Dorsey G (2006). Selection of Plasmodium falciparum pfmdr1 alleles following therapy with artemether-umefantrine in an area of Uganda where malaria is highly endemic. Antimicrob Agents Chemother.

[62] Nsobya SL, Dokomajilar C, Joloba M, Dorsey G, Rosenthal PJ (2007). Resistance-mediating Plasmodium falciparum pfcrt and pfmdr1 alleles after treatment with artesunate-amodiaquine in Uganda. Antimicrob Agents Chemother.

[63] Ochong E, Tumwebaze PK, Byaruhanga O, Greenhouse B, Rosenthal PJ (2013). Fitness consequences of Plasmodium falciparum pfmdr1 polymorphisms inferred from ex vivo culture of Ugandan parasites. Antimicrob Agents Chemother.

[64] Felger I, Snounou G, Hastings I, Moehrle JJ, Beck HP (2019). PCR correction strategies for malaria drug trials: updates and clarifications. Lancet Infect Dis.

[65] Plucinski MM, Morton L, Bushman M, Dimbu PR, Udhayakumar V (2015). Robust algorithm for systematic classification of malaria late treatment failures as recrudescence or reinfection using microsatellite genotyping. Antimicrob Agents Chemother.

[66] Jones S, Kay K, Hodel EM, Chy S, Mbituyumuremyi A, Uwimana A (2019). Improving methods for analyzing antimalarial drug efficacy trials: molecular correction based on length-polymorphic markers msp-1, msp-2, and glurp. Antimicrob Agents Chemother.

[67] Worldwide Antimalarial Resistance Network. ACT Partner Drug Molecular Surveyor. http://www.wwarn.org/tracking-resistance/act-partner-drug-molecular-surveyor. Accessed 28 Apr 2018.

[68] Okell LC, Reiter LM, Ebbe LS, Baraka V, Bisanzio D, Watson OJ (2018). Emerging implications of policies on malaria treatment: genetic changes in the Pfmdr-1 gene affecting susceptibility to artemether-lumefantrine and artesunate-amodiaquine in Africa. BMJ Glob Health.

[69] Griffin JT, Ferguson NM, Ghani AC (2014). Estimates of the changing age-burden of Plasmodium falciparum malaria disease in sub-Saharan Africa. Nat Commun.

[70] Hermsen CC, Telgt DS, Linders EH, van de Locht LA, Eling WM, Mensink EJ (2001). Detection of Plasmodium falciparum malaria parasites in vivo by real-time quantitative PCR. Mol Biochem Parasitol.

[71] Griffin JT, Hollingsworth TD, Okell LC, Churcher TS, White M, Hinsley W (2010). Reducing Plasmodium falciparum malaria transmission in Africa: a model-based evaluation of intervention strategies. PLoS Med.

[72] Plummer M (2013). rjags: Bayesian graphical models using MCMC. R package version 3–10.

[73] Therneau T (2015). A Package for Survival Analysis in S. version 2.38.

[74] Port GR, Boreham PFL, Bryan JH (1980). The relationship of host size to feeding by mosquitoes of the *Anopheles gambiae* Giles complex (Diptera: Culicidae). Bull Entomol Res.

[75] World Health Organization (2011). WHO anthro software, igrowup R package.

[76] WWARN Gametocyte Study Group (2016). Gametocyte carriage in uncomplicated *Plasmodium falciparum* malaria following treatment with artemisinin combination therapy: a systematic review and meta-analysis of individual patient data. BMC Med.

[77] Slater HC, Okell LC, Ghani AC (2017). Mathematical modelling to guide drug development for malaria elimination. Trends Parasitol.

[78] Falk N, Maire N, Sama W, Owusu-Agyei S, Smith T, Beck HP (2006). Comparison of PCR-rflp and genescan-based genotyping for analyzing infection dynamics of Plasmodium falciparum. Am J Trop Med Hyg.

[79] Yeka A, Kigozi R, Conrad MD, Lugemwa M, Okui P, Katureebe C (2016). Artesunate/amodiaquine versus artemether/lumefantrine for the treatment of uncomplicated malaria in Uganda: a randomized trial. J Infect Dis.

[80] The Global Fund (2018). Pooled procurement mechanism reference pricing: antimalarial medicines.

[81] Pfeil J, Borrmann S, Tozan Y (2014). Dihydroartemisinin-piperaquine vs. artemether-lumefantrine for first-line treatment of uncomplicated malaria in African children: a cost-effectiveness analysis. PLoS One.

[82] Coldiron ME, Von Seidlein L, Grais RF (2017). Seasonal malaria chemoprevention: successes and missed opportunities. Malar J.

[83] http://www.tropmedres.ac/trac-ii-2. Accessed 4 Mar 2018.

[84] Pongtavornpinyo W, Hastings IM, Dondorp A, White LJ, Maude RJ, Saralamba S (2009). Probability of emergence of antimalarial resistance in different stages of the parasite life cycle. Evol Appl.

[85] Kay K, Hastings IM (2015). Measuring windows of selection for anti-malarial drug treatments. Malar J.

