# Supplementary material for: The duration of chemoprophylaxis against malaria after treatment with artesunate-amodiaquine and artemether-lumefantrine and the effects of pfmdr1 86Y and pfcrt 76T: a meta-analysis of individual patient data
Source: BMC Med. 2020 Feb 25;18:47. doi: 10.1186/s12916-020-1494-3 (PMC7043031; doi:10.1186/s12916-020-1494-3)
Supplement: Supplementary file 1 — Additional file 1: Table S1. Risk factors for reinfection: multivariable analysis with pfcrt 76T. Data from AS-AQ and AL trial arms were analyzed separately using accelerated failure-time analysis. Regression coefficients are the ratio of time to reinfection, such that a coefficient > 1 indicates a longer time to reinfection. Covariates significantly associated with reinfection time after adjusting for EIR (Table 3, main text) were included in the final model. The prevalence of pfmdr1 86Y also had a significant effect in a multivariable model with the same covariates (Table 3, main text) but could not be included in the same model with pfcrt 76T due to strong correlation between the two variables. Models assume a log-normal time to reinfection and random site effects. [file 12916_2020_1494_MOESM1_ESM.docx]

|  | AL multivariable model (N=1724):  EIR, age, dose, *pfcrt1* 76T | | AS-AQ multivariable model (N=1998)  EIR, age, *pfcrt1* 76T | |
| --- | --- | --- | --- | --- |
| Covariate (unit) | Coefficient [ratio of reinfection times] (95% CI) | P value | Coefficient [ratio of reinfection times] (95% CI) | P value |
| Log_e_ EIR | 0.80 (0.74, 0.87) | <0.001 | 0.81 (0.74, 0.87) | <0.001 |
| age (years, >20 grouped together) |  | <0.001 |  | <0.001 |
| age  (age)^2^  (age)^3^ | 1.02 (0.94, 1.11)  1.00 (0.98, 1.02)  1.0001 (0.9993, 1.0010) |  | 0.94 (0.886, 1.00)  1.01 (1.00, 1.02)  0.9997 (0.9992, 1.0001) |  |
| Lumefantrine dose (per 10 mg per kg increase) (in AL arms only) | 1.03 (1.01, 1.05) | 0.002 | - |  |
| *pfmdr* 76T prevalence (per 10% increase) | 1.04 (1.01, 1.07) | 0.005 | 0.98 (0.95, 1.01) | 0.162 |
