# Supplementary figures and images for: The duration of chemoprophylaxis against malaria after treatment with artesunate-amodiaquine and artemether-lumefantrine and the effects of pfmdr1 86Y and pfcrt 76T: a meta-analysis of individual patient data
Source: BMC Med. 2020 Feb 25;18:47. doi: 10.1186/s12916-020-1494-3 (PMC7043031; doi:10.1186/s12916-020-1494-3)

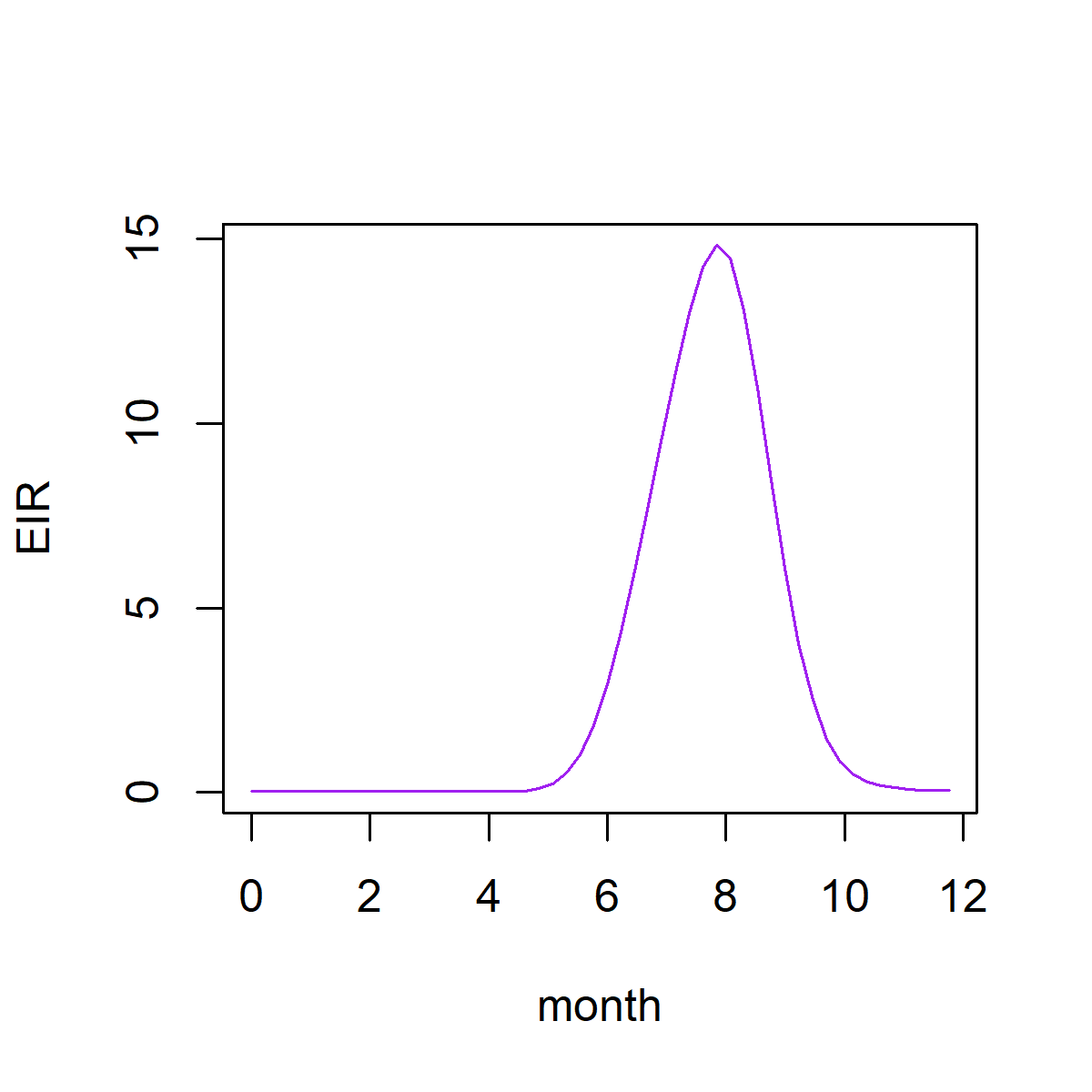

Supplement: Supplementary file 2 — Additional file 2:Figure S1. Simulated annual seasonal variation in EIR assumed in the analysis of potential impact of AL and AS-AQ on population level transmission (Fig. 5, main text). The EIR shown is for the simulated seasonal medium transmission setting (slide prevalence = 15%), but the relative EIR variation across the year was the same in the seasonal low and high simulated transmission settings. [file 12916_2020_1494_MOESM2_ESM.tiff]

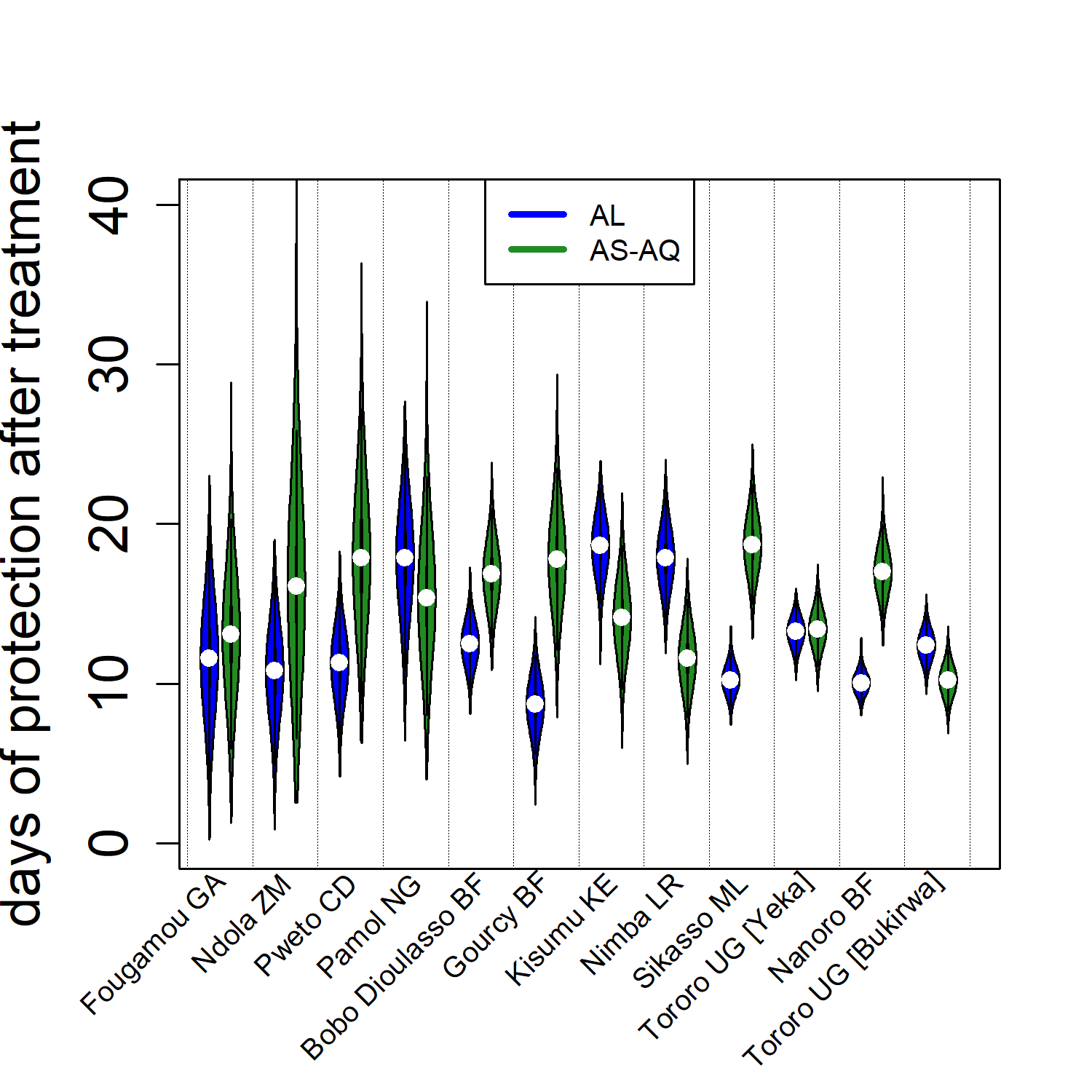

Supplement: Supplementary file 3 — Additional file 3: Figure S2. The duration of post-treatment prophylaxis at different trial locations in order of increasing estimated EIR. Posterior estimates of the duration of protection provided by AL or AS-AQ are shown. The study sites are shown in order of increasing transmission intensity left to right according to posterior EIR estimates. [file 12916_2020_1494_MOESM3_ESM.tiff]

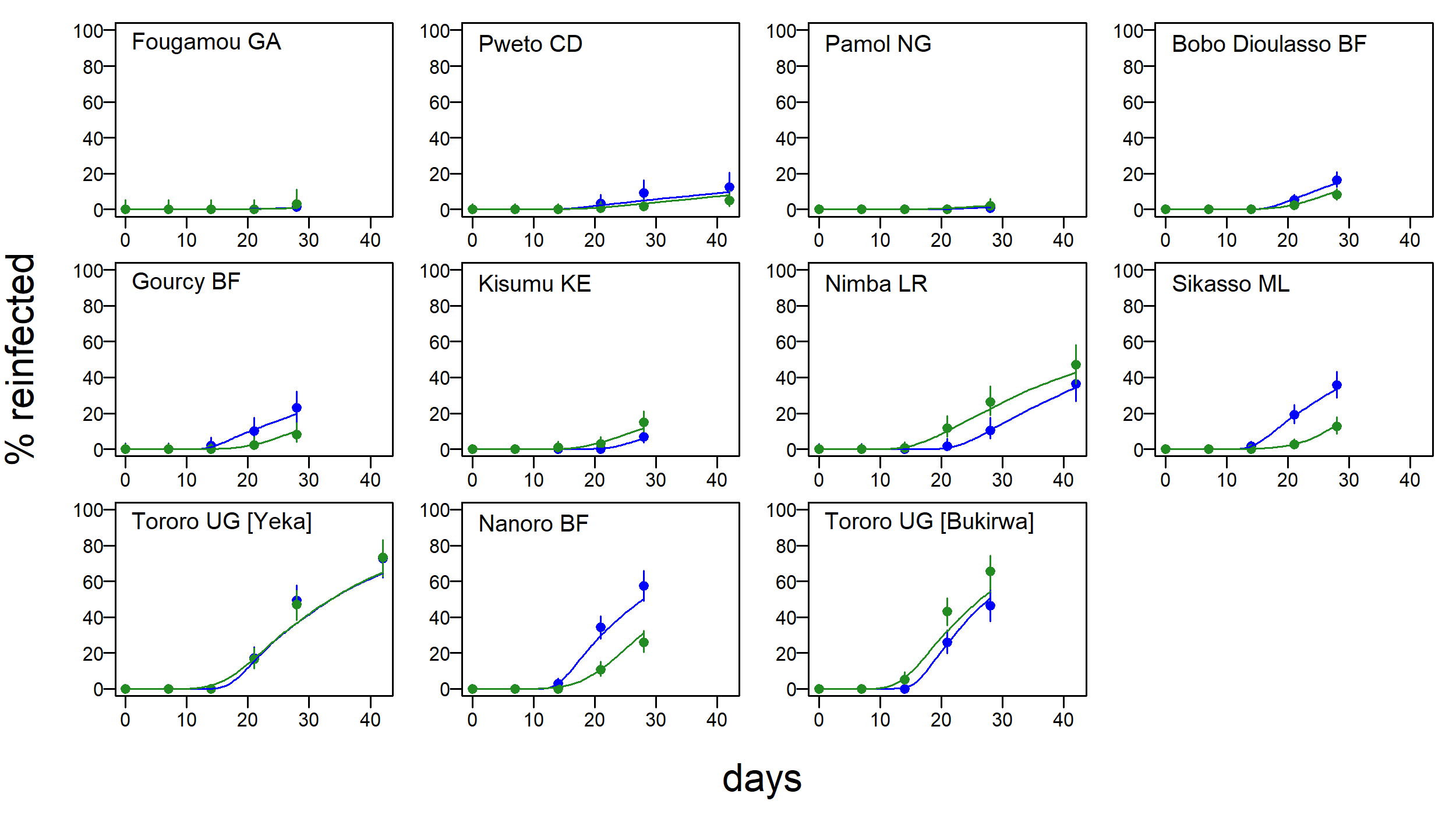

Supplement: Supplementary file 4 — Additional file 4: Figure S3. Sensitivity analysis of PCR-correction misclassification: time to reinfection after treatment and model fits. Here we repeated the analysis shown in Fig. 2 of the main text on a modified dataset, in which we explored the impact of reclassifying some reinfections as recrudescences and removing them from the analysis. To reclassify reinfections for each trial arm, we sampled a number of reinfections equal to 3% of the study population, with probability weighted according to the estimated timings of recrudescence in Fig. 5 of [66] and the relative frequency of apparent ‘reinfection’ timing in the current dataset (such that the probabilities of sampling of reinfections, if present, at days 7,14,21,28,35, and 42 were 0.799, 0.100, 0.026, 0.014, 0.049, and 0.012). One site, Ndola in Zambia, was excluded after reclassification of reinfections, since one trial arm no longer contained reinfections and the model could not be fitted. The Figure shows the proportion of patients reinfected during follow up, amongst patients not experiencing recrudescence, after treatment at day 0 with AL (blue) or AS-AQ (green) in each of the 11 trial sites included in this sensitivity analysis. Circles show data with 95% CI, and the lines are the fits of the hidden semi-Markov model in each site. Here, the AL trial arms include in total 1956 individuals, 573 reinfections, and the AS-AQ trial arms, 2001 individuals, 475 reinfections. [file 12916_2020_1494_MOESM4_ESM.tiff]

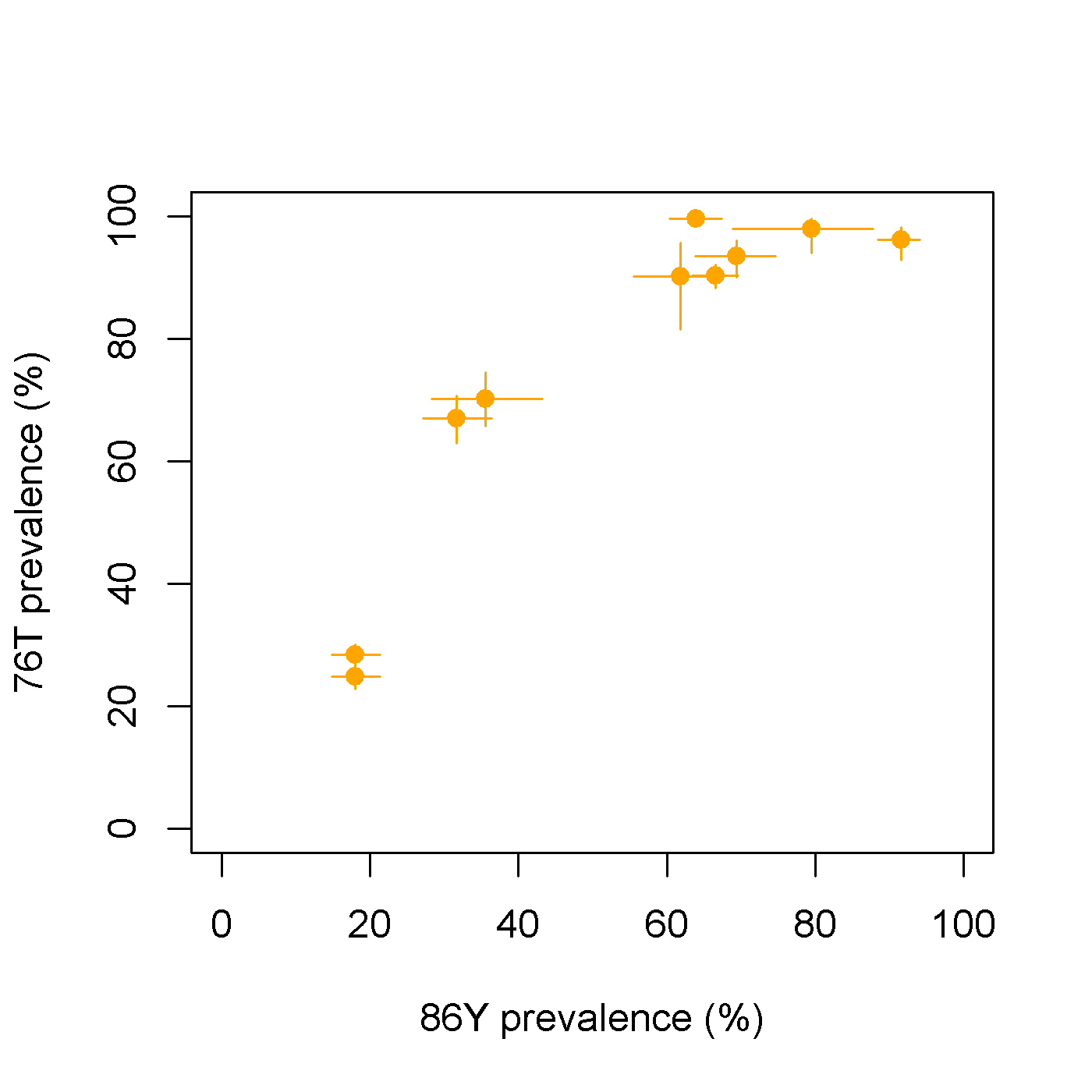

Supplement: Supplementary file 5 — Additional file 5: Figure S4. Correlation between pfcrt 76T prevalence and pfmdr 86Y prevalence, in the surveys matched to the trial sites according to year and geographic distance (within 1 year and 300 km in the same country as each trial). When more than one molecular marker survey was matched to a trial site, a weighted average prevalence was taken. In some cases, these two molecular markers were assessed in the same matched survey(s), but in other cases matches from different surveys were found. [file 12916_2020_1494_MOESM5_ESM.tiff]

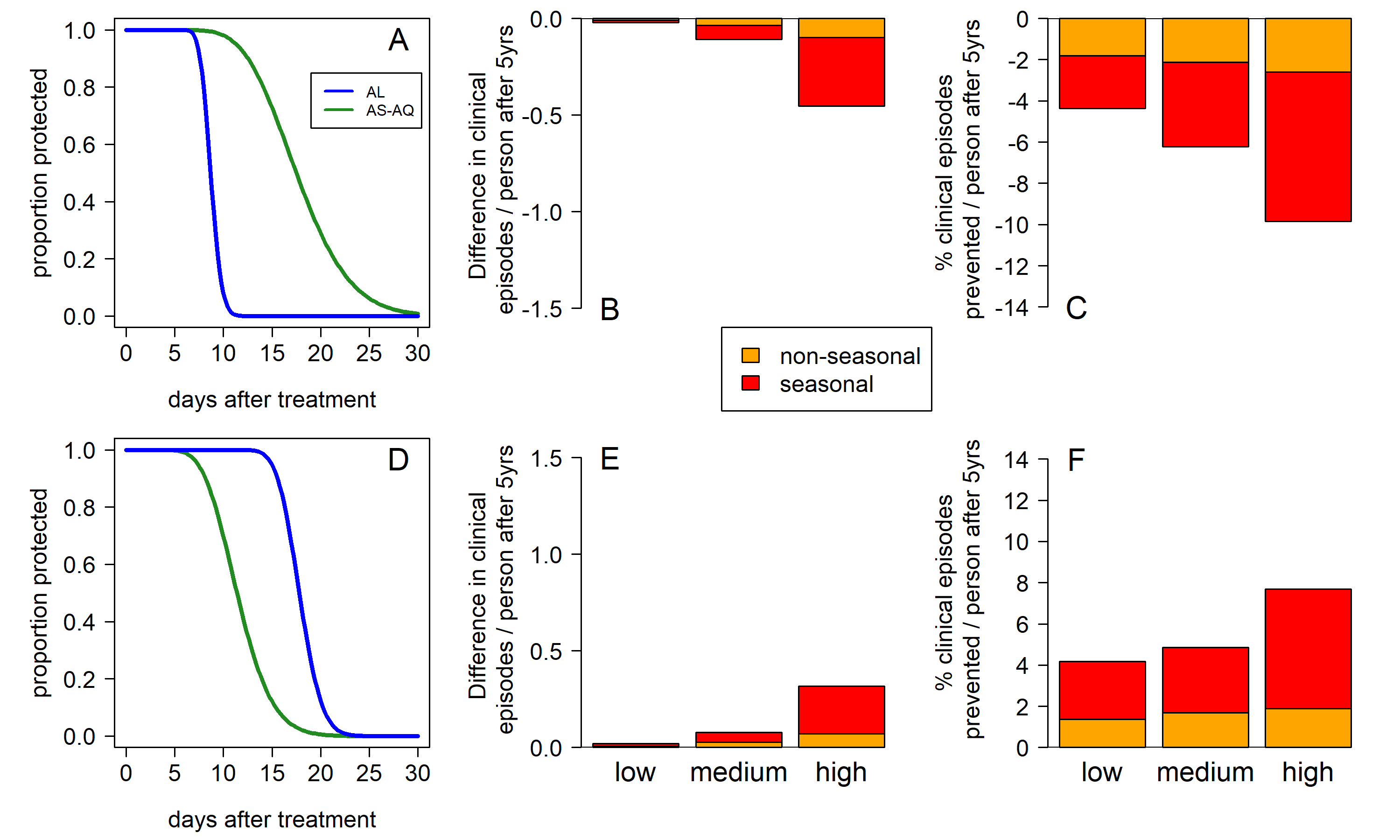

Supplement: Supplementary file 6 — Additional file 6: Figure S5. As Fig. 5 in the main text, except panels B,C,E and F show impact on clinical incidence in the whole population (rather than 0–5 year old children only). [file 12916_2020_1494_MOESM6_ESM.tiff]
